# Gentamicin and antibiotic use in Norwegian hospitals: a study from 35 point-prevalence surveys, 2015 to 2023

**DOI:** 10.2807/1560-7917.ES.2026.31.42.2500969

**Published:** 2026-10-22

**Authors:** Marta Vuković, Siri Nordtveit, Brita Skodvin, Katerina Nezvalova-Henriksen, Hege Salvesen Blix, Jannicke Slettli Wathne, Kristian Tonby

**Affiliations:** 1 Department of Clinical Pharmacy and Advisory Services, Oslo Hospital Pharmacy, Oslo, Norway; 2 Department of Infectious diseases, Oslo University Hospital; 3 Institute of Clinical Medicine, Faculty of Medicine, University of Oslo, Oslo, Norway; 4 Norwegian Centre for Antibiotic Use in Hospitals, Bergen, Norway; 5 Department of Research and Development, Haukeland University Hospital, Bergen, Norway; 6 Antibiotic Resistance and Infection Prevention, Norwegian Institute of Public Health, Oslo, Norway

**Keywords:** Gentamicin, Point-prevalence survey, Antibiotic stewardship, Aminoglycosides, Antimicrobial resistance (AMR), Hospital antibiotic use, WHO AWaRe, COVID-19

## Abstract

**BACKGROUND:**

Inappropriate and excessive use of broad-spectrum antibiotics drives antimicrobial resistance. Gentamicin, an Access antibiotic in the WHO AWaRe framework with low resistance potential, has long been recommended in Norway for empirical treatment of serious infections. However, real-world data on aminoglycoside use remain limited.

**AIM:**

We aimed to describe gentamicin and overall antibiotic use in Norway, including COVID-19 pandemic-related changes, and report the extent of gentamicin use across selected indications.

**METHODS:**

Data were obtained from patients ≥ 20 years from 35 national point-prevalence surveys (2015–2023) across 68 Norwegian hospitals.

**RESULTS:**

Among 241,546 hospitalised patients, 66,586 received antibiotics (yearly prevalence 26.9–28.1%), with the highest proportions in the 70–79 (26.4%) and ≥ 80 (23.4%) years age groups. Aminoglycosides, mainly gentamicin, ranked ninth among all antibiotic prescriptions. From 2021, third-generation cephalosporins (13–14%) surpassed β-lactamase–sensitive penicillins (12–13%). Gentamicin use increased from 4.1% in 2015 to 5.8% in 2023, mainly in combination with ampicillin, benzylpenicillin, or ampicillin plus metronidazole. Main indications were urinary tract, lower respiratory tract and intraabdominal infections, and sepsis. An increasing pre-COVID-19 pandemic trend in gentamicin use (β_1_: 0.105; p < 0.001) was no longer observed after the COVID-19 outbreak was declared a global pandemic on the 11 March 2020 (β_3_:  − 0.112; p = 0.013).

**CONCLUSION:**

Gentamicin use among hospitalised patients in Norway remains low but appears higher than in other European countries. Increases in gentamicin use pre-COVID-19 pandemic were no longer observed after COVID-19, supporting the need for surveillance and antibiotic stewardship to minimise unnecessary use of broad-spectrum antibiotics.

**Key public health message**

**What did you want to address in this study and why?**

Overuse of broad-spectrum antibiotics drives antimicrobial resistance (AMR). Surveillance data on antibiotic use can help identify areas for improvement. Gentamicin is recommended in Norway for treating severe infections and is considered less likely to drive AMR than many broad-spectrum antibiotics. We wanted to examine how gentamicin and other antibiotics are used in Norwegian hospitals and whether these changed during the COVID-19 pandemic.

**What have we learnt from this study?**

Overall antibiotic use remained stable and was lower than in other European countries. Gentamicin use increased slightly but remained relatively low despite its important role in treatment guidelines. However, the use of broad-spectrum antibiotics increased from 2021. The pattern of gentamicin use also changed after 2020.

**What are the implications of your findings for public health?**

Regular monitoring of antibiotic use can identify where prescribing practices could be improved. We identified areas for improvement, including increasing gentamicin use and reducing reliance on broad-spectrum antibiotics. Improving surveillance and comparing prescribing practices between countries could help hospitals use antibiotics more appropriately and limit AMR.

## Introduction

Antimicrobial resistance (AMR) is a major global health threat [1], driven largely by inadequate and overuse of antimicrobials [2]. Surveillance data on AMR and antimicrobial use are indispensable for comparing consumption patterns at local, national and global settings [3]. Point-prevalence surveys (PPS) are a recommended approach for capturing patient-level antimicrobial use at specific time points and may include clinical and demographic data [4], thereby providing more detailed insight into prescribing practices and treatment indications.

Aminoglycosides, including gentamicin, are potent bactericidal antibiotics, mainly used against Gram-negative pathogens, typically in combination therapy [5]. Gentamicin is considered to have a lower potential to drive AMR and exerts limited collateral damage on the gut microbiome [6]. It is classified as an Access antibiotic within the World Health Organization (WHO) AWaRe (Access, Watch, Reserve) framework, which guides antibiotic stewardship. Access antibiotics are preferred for common infections, Watch antibiotics carry a higher risk of driving resistance and Reserve antibiotics are reserved for last-line treatment for multidrug-resistant infections [7].

Across Europe, aminoglycosides are used as add-on therapy to broad-spectrum antibiotics in the treatment of serious infections and have regained attention as treatment options for infections caused by multidrug-resistant Gram-negative bacilli [8,9]. Resistance rates to aminoglycosides are favourable in Norway [10,11], a country with a low prevalence of AMR compared with other European countries. For decades, aminoglycosides such as gentamicin, in combination with narrow-spectrum antibiotics, have been recommended in Norway as first-line empirical treatment for sepsis and other serious infections [12]. Nevertheless, real-world data on aminoglycoside use in clinical practice remain scarce. Furthermore, the COVID-19 pandemic has been associated with changes in antimicrobial prescribing [13], but data on its impact remain limited.

In this study, we draw on data from large national PPS conducted between 2015 and 2023 to describe the patterns of gentamicin and overall antibiotic use and to assess the effect of COVID-19 on gentamicin and across AWaRe categories. We also evaluate the extent of gentamicin use and the clinical indications for its use in the treatment of serious infections among adult hospitalised patients.

## Methods

### Study design and data collection

Data were derived from 35 cross-sectional PPS conducted in Norwegian hospitals between May 2015 and November 2023. Since May 2015, hospitals have been required to participate in twice-yearly PPS in May and November, with additional voluntary surveys in February and September, scheduled by the Norwegian Institute of Public Health (NIPH). Our study included all available mandatory and voluntary PPS over this 9-year period, excluding long-term care facilities. In total, 68 hospitals across Norway contributed data and all hospital data were analysed anonymously.

Health professionals, either working on the wards or in infection control departments, recorded data in the data collection protocol from NIPH as appended in the Data Collection Template (Supplementary Material). In our study, data were collected from patients (≥ 20 years) admitted by 08:00 on the day of the surveys across all hospital departments, except for psychiatric and addiction units. The age cut-off at 20 years and the 10-year age group intervals of 20–29, 39–39, 40–49, 50–59, 60–69, 70–79 and 80 years and older are due to the predefined categories described in the Supplementary Material Data Collection Template.

The collected data were stored in the national registry of the NIPH. Data collection, storage and management are governed by the Regulation on Norwegian Surveillance System for Antibiotic Use and Healthcare-Associated Infections [14].

### Norwegian healthcare system and national guidelines

Four state-owned regional health authorities are responsible for organising and delivering specialist healthcare services in Norwegian hospitals. In total, Norway has 74 hospitals, including fewer than 10 small private institutions [15].

National guidelines for antibiotic use in hospitals were published in 2013. These guidelines are normative, publicly available and regularly updated by the Norwegian Directorate of Health [12]; implementation is the responsibility of each hospital.

Continuous revisions of the guidelines have been undertaken since 2020 [16]. Supplementary Table S1 provides an overview of the relevant guideline recommendations and demonstrates the continued central role of gentamicin in first-line empirical treatment of severe infections throughout the study period.

### Data preparation and statistical analysis

Patients were included in the analysis based on the following criteria: the patient had been assigned a unique de-identified numerical code and was receiving treatment for a single indication. Patients receiving more than three antibiotics were excluded from the analysis since Norwegian guidelines do not recommend treatment with more than three antibiotics for any of the specific indications.

First, a prescription-level analysis included all antibiotic prescriptions during the study period, allowing assessment of overall prescribing patterns. Second, a patient-level analysis aggregated prescriptions for each patient, enabling evaluation of treatment regimens, including combinations of antibiotics and their use across specific indications at the individual level. Patients were categorised according to whether they received gentamicin prescription during the study period (‘gentamicin-treated’) or no gentamicin prescription. Dosing was not analysed due to a lack of information on patients’ weight, which was needed to assess aminoglycoside dosing.

Data preprocessing was performed using IBM SPSS Statistics version 18 (IMB Corp, Armonk, United States (US)). All remaining analyses were performed in STATA version 17 (StataCorp LLC, College Station, Texas, US). Differences in patient characteristics between the ‘gentamicin-treated’ versus ‘no gentamicin’ groups were analysed using Pearson’s chi-square test. Interrupted time series (ITS) analysis [17] was used to analyse the effect of the COVID-19 pandemic on gentamicin use and overall antibiotics use according to the AWaRe categories. Antibiotic class and substances prescribed in study population according to AWaRe categories are presented in Supplementary Table S2. The Watch and Reserve categories were combined due to low use of Reserve antibiotics in Norway. Statistical significance was defined as p < 0.05 for all analyses.

## Results

### Study population

A total of 68 hospitals participated between 2015 and 2023, with generally high participation across the study period (median 55 hospitals per survey; interquartile range (IQR):21), as shown in Supplementary Figure S1A.

Overall, 66,586 patients were treated with 78,356 antibiotics between 2015 and 2023 (Figure 1). Supplementary Table S3 shows that the prevalence of patients receiving antibiotics remained stable during the study period, ranging between 26.9 and 28.1%. Across surveys, the median number of patients treated with antibiotics was 2,230 (IQR: 938), with moderate variability and no obvious outliers as presented in Supplementary Figure S1B.

**Figure 1:**
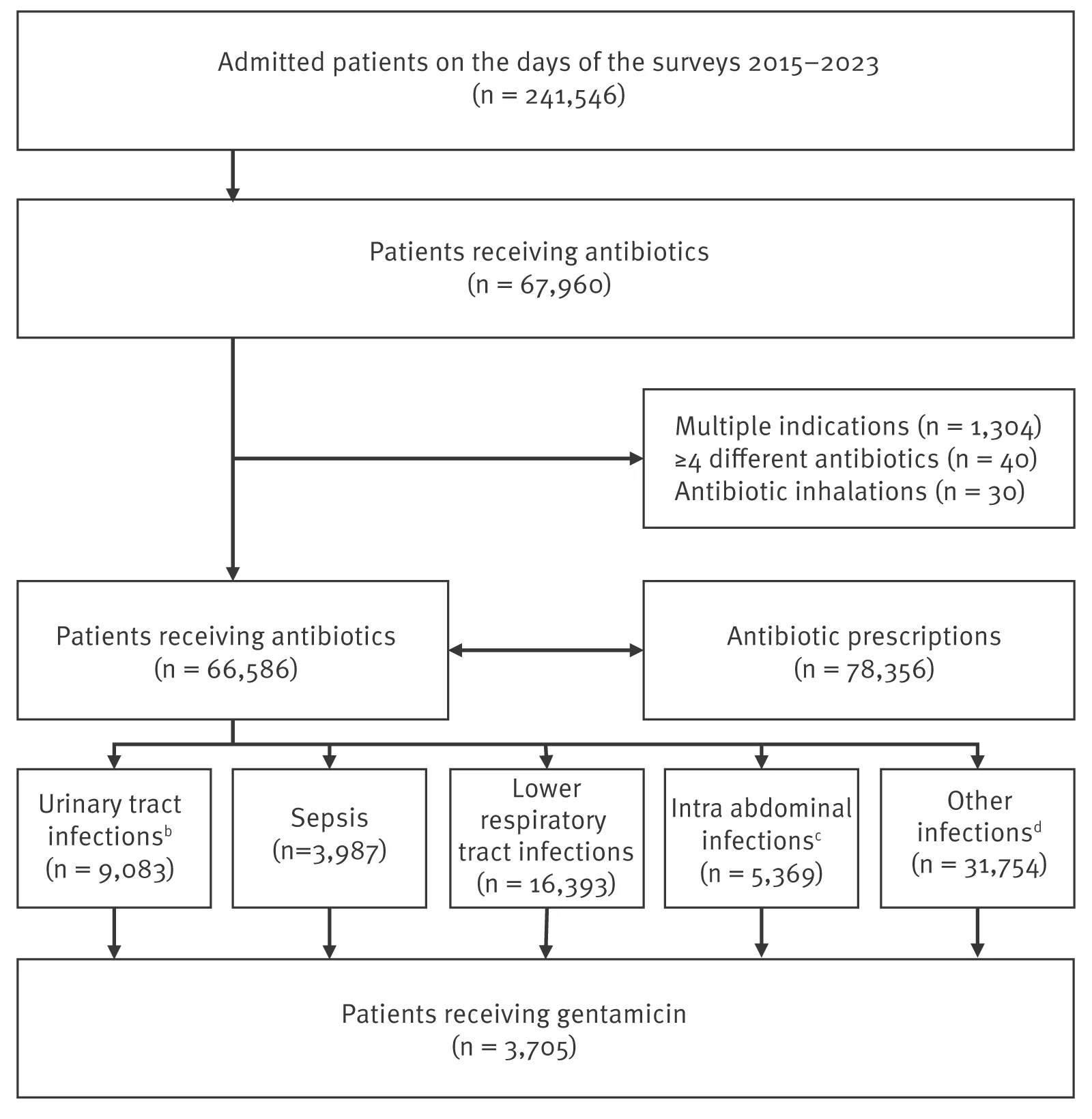
Flowchart of study inclusion of hospitalised adults^a^ receiving antibiotics, Norway, 2015–2023 (n = 66,586 patients; n = 78,356 prescriptions)

^a^ Included adults were 20 years old or older.

^b^ Urinary tract infections (UTI) include both upper and lower UTI.

^c^ Post-operative intraabdominal infections excluded.

^d^ Other Infections include post-operative infections (n = 10,459), skin and soft tissue infections (n = 5,194), bone and joint infections (n = 2,680), cardiovascular infections (n = 1,267), febrile neutropenia (n = 989), other infections (n = 2,417), unknown (n = 8,748).

Most patients were older adults, with the highest proportions in the 70–79 years (26.4%) and 80 years and older (23.4%) age groups. Among participants, 53.9% were male, 46.1% female and 0.1% of unknown sex. Detailed demographic data for all patients receiving antibiotics, including gentamicin, are shown in the Table. Overall, there were significant differences in the main baseline characteristics between the groups, including sex, age, type of indication and department speciality. The highest proportion of gentamicin-treated patients (total n = 3,705) was found in the 70–79 years age group (26.3%). Community-acquired infections were the most frequent indication overall (57.4%) and were especially frequent among gentamicin recipients (78.9%).

**Table:** Study population receiving antibiotics, Norway, 2015–2023 (n = 66,586)

Baseline characteristics	Total (n = 66,586)		No gentamicin (n = 62,881)		Gentamicin-treated (n = 3,705)		p value^a^
	n	%	n	%	n	%	
**Sex**							
Male	35,868	53.9	33,800	53.8	2,068	55.8	0.032
Female	30,672	46.1	29,036	46.2	1,636	44.2	
Unknown	46	0.1	45	0.1	1	0.0	
**Age (years)**							
20–29	2,645	4.0	2,456	3.9	189	5.1	< 0.001
30–39	3,424	5.1	3,179	5.1	245	6.6	
40–49	4,551	6.8	4,260	6.8	291	7.9	
50–59	7,829	11.8	7,393	11.8	436	11.8	
60–69	12,897	19.4	12,167	19.3	730	19.7	
70–79	17,611	26.4	16,638	26.5	973	26.3	
≥ 80	15,560	23.4	14,838	23.6	722	19.5	
Unknown	2,069	3.1	1,950	3.1	119	3.2	
**Department specialty**							
Surgery	23,371	35.1	22,347	35.5	1,024	27.6	< 0.001
Internal medicine	29,334	44.1	27,304	43.4	2,030	54.8	
Obstetrics and gynaecology	1,910	2.9	1,798	2.9	112	3.0	
Oncology	2,095	3.1	1,944	3.1	151	4.1	
Intensive care unit	5,608	8.4	5,369	8.5	239	6.5	
Other departments	4,268	6.4	4,119	6.6	149	4.0	
**Type of indication**							
Healthcare-associated infection	12,000	18.0	11,501	18.3	499	13.5	< 0.001
Surgical or medical prophylaxis	13,101	19.7	12,990	20.7	111	3.0	
Community-acquired infection	38,214	57.4	35,290	56.1	2,924	78.9	
Unknown	3,271	4.9	3,100	4.9	171	4.6	
**Number of antibiotics per patient**							
1	55,816	83.8	55,626	88.5	190	5.1	< 0.001
2	9,770	14.7	6,918	11.0	2,852	77.0	
3	1,000	1.5	337	0.5	663	17.9	
**Indication documented in the patient records**							
Yes	54,805	82.3	51,453	81.8	3,352	90.5	< 0.001
No	2,231	3.4	2,166	3.4	65	1.8	
Unknown	9,550	14.3	9,262	14.7	288	7.8	

^a^ The difference between groups was evaluated using Pearson’s chi-square test.

### Overall patterns of antibiotic use, 2015–2023

First, we performed prescription-level analysis of all antibiotic prescriptions (n = 78,356). β-lactamase–sensitive penicillins constituted the largest prescription class (Figure 2); their use increased yearly until 2019 to 15.0% and declined to 12.9% by 2023. Supplementary Table S4 shows that benzylpenicillin comprised 87.6% of prescriptions within this class during 2015–2023, while third- and higher-generation cephalosporins consisted mainly of cefotaxime (84.5%) and ceftriaxone (11.9%). Detailed prescription patterns during study period are presented in Supplementary Table S5. From 2021, third- and higher-generation cephalosporins surpassed β-lactamase-sensitive penicillins and remained higher throughout (14.1% in 2021 and 2022 and 13.4% in 2023 vs 12.6%, 11.7% and 12.9%, respectively), indicating a sustained shift towards broader-spectrum use.

**Figure 2:**
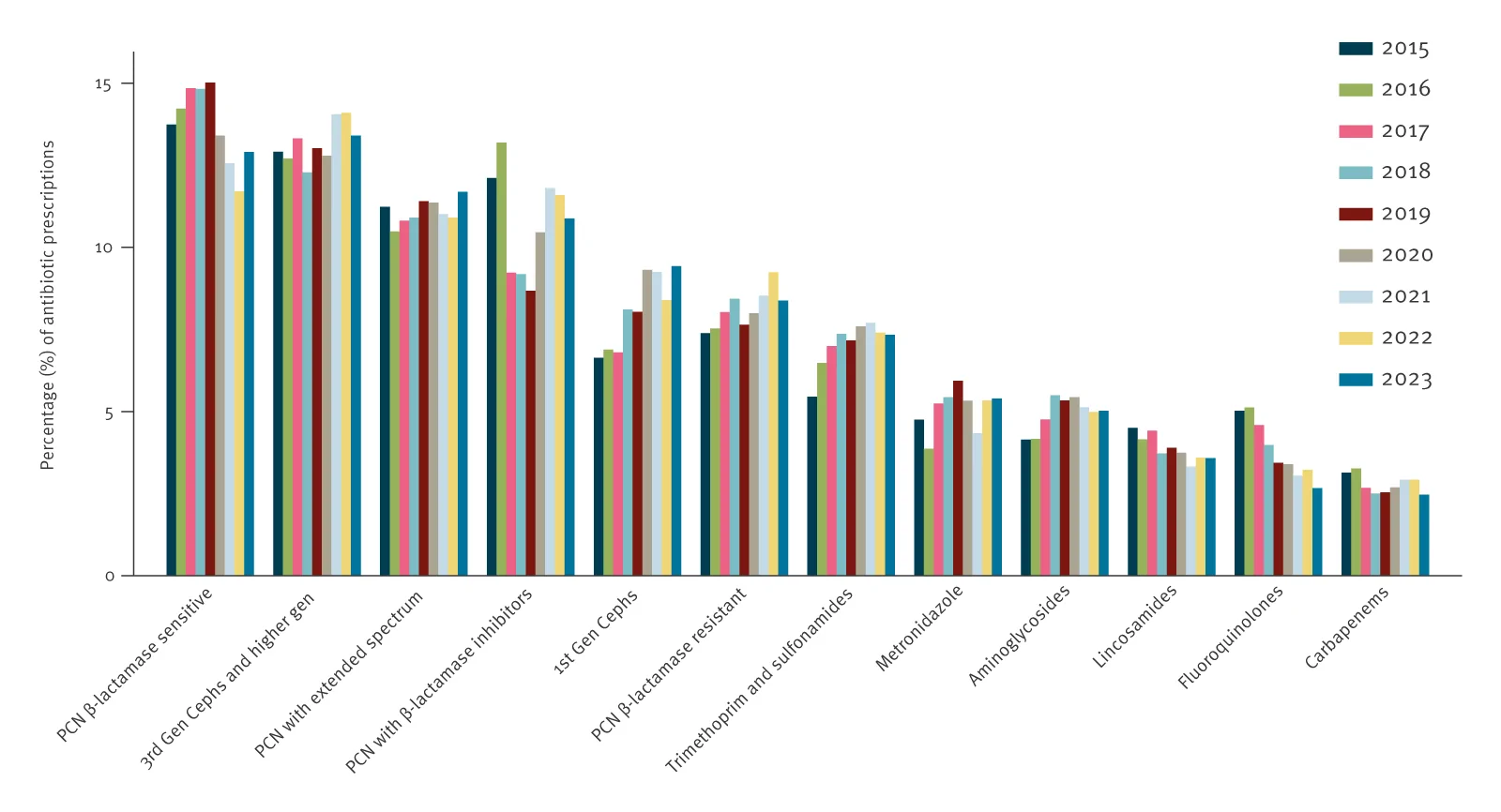
Distribution of the 12 largest prescription groups (shown as a percentage of all prescriptions) registered in the point-prevalence surveys in Norwegian hospitals, 2015–2023 (n = 78,356)

Cephs: cephalosporins; gen: generation; PCN: penicillins.

See Supplementary Table S4 for an overview of antibiotic substances in each Anatomical Therapeutic Chemical group and Supplementary Table S5 for a detailed overview of prescriptions.

Supplementary Table S5 shows that extended-spectrum penicillins accounted for ca 11.0% of prescriptions during the study period, while the frequency distribution in Supplementary Table S4 shows that ampicillin (43.7%) and amoxicillin (29.4%) were the most frequently prescribed substances within the Anatomical Therapeutic Chemical (ATC) group. Supplementary Table S5 further shows that the use of penicillins with β-lactamase inhibitors, primarily piperacillin/tazobactam (97.3%), declined from 13.2% in 2016 to ca 9.0% during 2017–2019 and was 10.9% in 2023.

At the prescription level, among all antibiotic classes analysed, aminoglycosides were the ninth most frequently prescribed class, with an increase from 4.1% in 2015 to 5.0% in 2023, shown in Supplementary Table S5. Gentamicin accounted for 95% of all aminoglycoside prescriptions, while tobramycin and amikacin were rarely used; numbers are presented in Supplementary Table S4.

### Effect of the COVID-19 pandemic on gentamicin and antibiotic use according to AWaRe categories

The effect of the COVID-19 pandemic on antibiotic use according to the WHO AWaRe categories was assessed at the prescription level (n = 78,356) with interrupted times series analysis (Figure 3).

**Figure 3:**
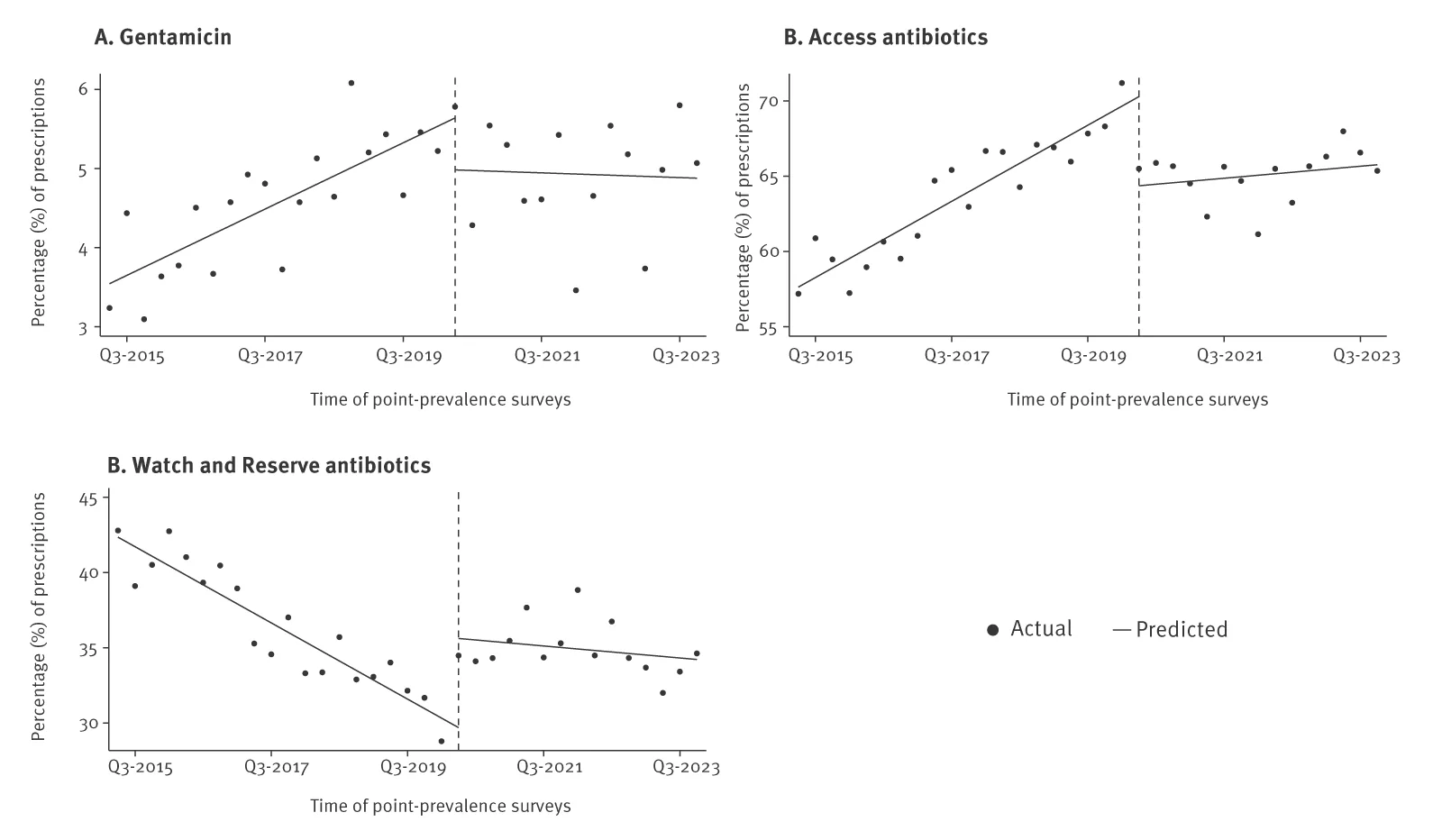
Use of gentamicin and overall antibiotics according to the World Health Organization AWaRe categories before and after the COVID-19 pandemic, with interrupted time series trend lines, Norway, 2015–2023 (n = 78,356)

Q: quarter.

The dashed line marks the first point-prevalence survey after the start of the COVID-19 pandemic (Q2 2020).

Gentamicin was also included in the Access category of antibiotics.

Please see Supplementary Table S6 for statistical analyses.

Interrupted time series analysis demonstrated significant changes in antibiotic use following the onset of the COVID-19 pandemic in Norway (second quarter (Q2) 2020). Before the COVID-19 pandemic, gentamicin use increased significantly over time (β_1_: 0.105 percentage points per quarter; p < 0.001, confidence intervals are presented in Supplementary Table S6). No significant immediate change was observed after Q2 2020, but the post-pandemic trend shifted significantly towards a slight decline (change in slope β_3_:  − 0.112 percentage points per quarter; p = 0.013) as shown in Supplementary Table S6).

Access antibiotics showed a significant increasing trend before the pandemic (β_1_: 0.632 percentage points per quarter; p < 0.001), followed by a significant immediate decline in level after Q2 2020 (β_2_:  − 5.920; p < 0.001) and a subsequent flattening of the trend (change in slope β_3_:  − 0.533; p < 0.001). In contrast, Watch/Reserve antibiotics demonstrated a significant pre-pandemic decline followed by a significant increase in level and a change in trend after the onset of the COVID-19 pandemic. All values including confidence intervals are presented in Supplementary Table S6.

### Low but increasing use of gentamicin combinations

To assess gentamicin prescribing at the individual patient level, including antibiotic combinations, we conducted a patient-level analysis of gentamicin use among all patients receiving antibiotics (Figure 4).

**Figure 4:**
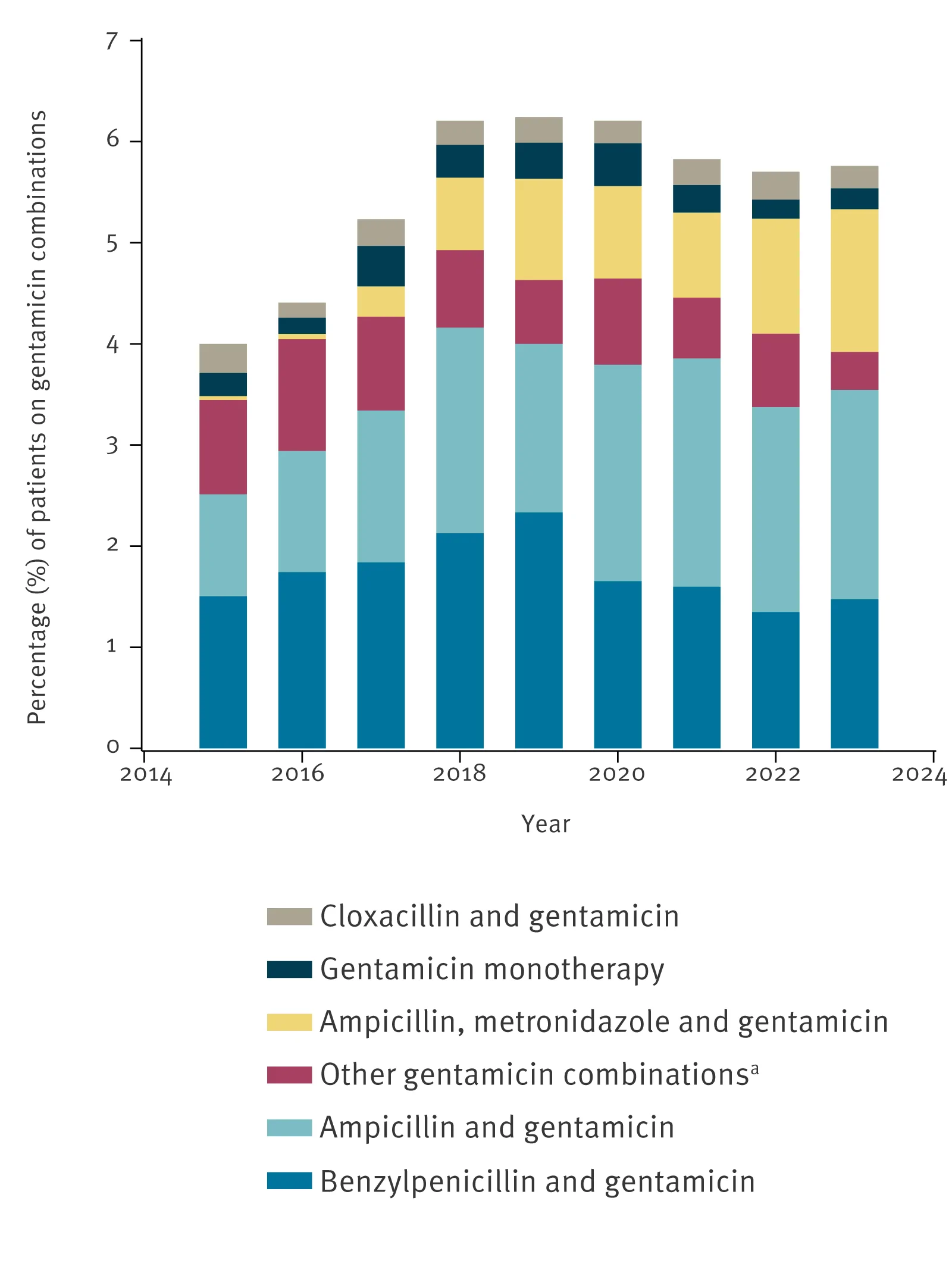
Distribution of gentamicin combinations among all patients receiving antibiotics, Norway, 2015–2023 (n = 66,586)

^a^ Other gentamicin combinations include gentamicin in combination with one of the following substances: clindamycin, metronidazole, cephalothin, amoxicillin, ciprofloxacin, cefotaxime, vancomycin and piperacillin/tazobactam.

See Supplementary Table S3 for details of different gentamicin combinations during the study period.

The proportion of patients treated with gentamicin combinations increased from 4.0% in 2015 to 5.8% in 2023 (Figure 4). Detailed annual numbers for the most common gentamicin-containing combinations are provided in Supplementary Table S3. The most common treatment combinations were benzylpenicillin and gentamicin, ampicillin and gentamicin, and ampicillin, gentamicin and metronidazole. The use of benzylpenicillin in combination with gentamicin increased from 1.5% in 2015 to 2.3% in 2019 and declined to 1.5% in 2023. Treatment with ampicillin, gentamicin and metronidazole showed the most pronounced increase, from 0.0% in 2015 to 1.4% in 2023. Gentamicin in combination with ampicillin increased from 1.0% in 2015 to 2.0% in 2018 and remained at ca 2% throughout the study period.

### Patterns of gentamicin use across major infection sites and sepsis

Among gentamicin recipients (n = 3,705), the most frequent indications were urinary tract infections (UTI), including both lower and upper UTI, sepsis, lower respiratory tract infections (LRTI) and intraabdominal infections (IAI), presented in Supplementary Figure S2. Consequently, all patients receiving antibiotics for these indications were included in the subsequent analysis (Figure 5A–D). 

**Figure 5:**
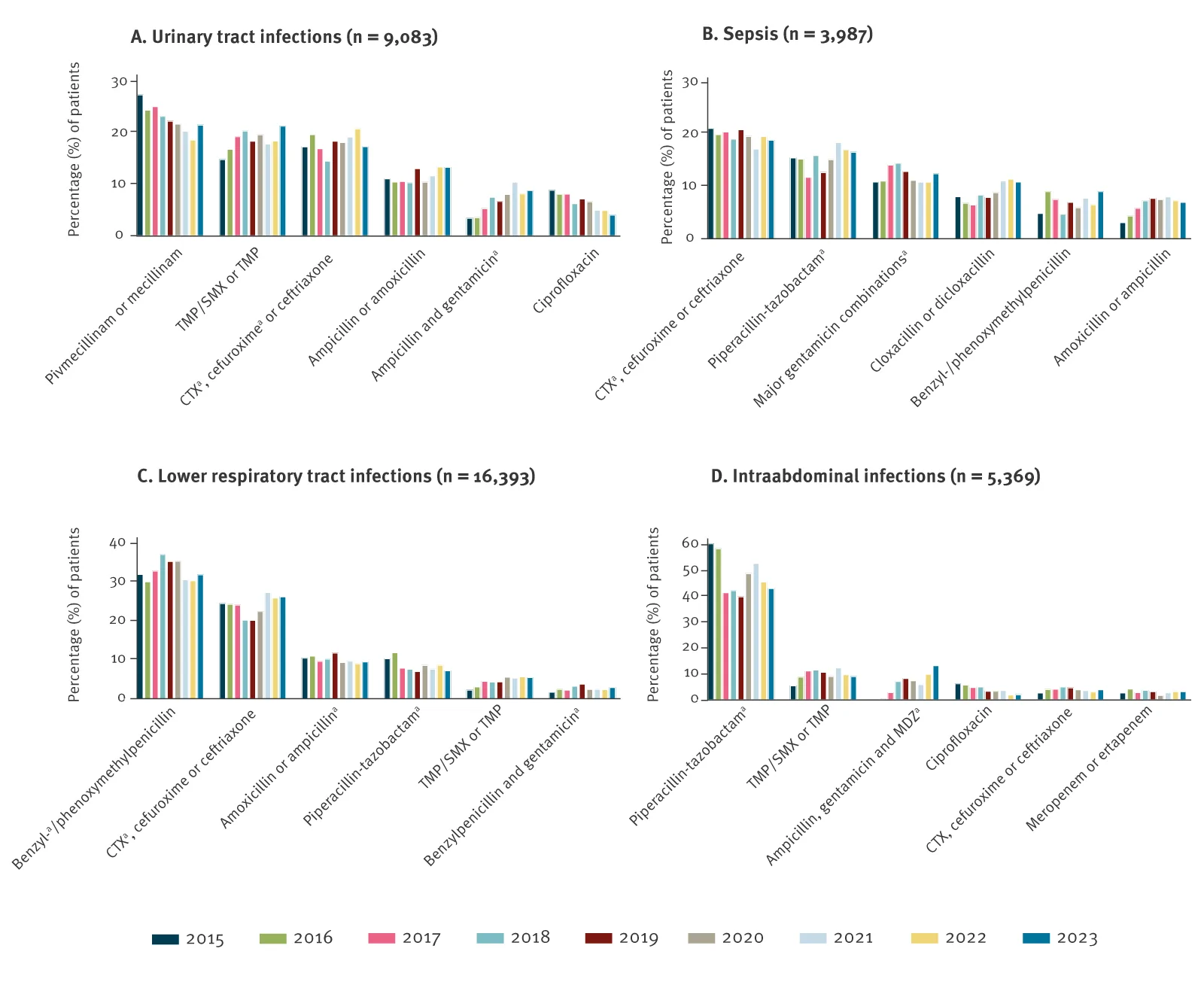
Distribution of major antibiotic regimens, including gentamicin combinations, in hospitalised patients on antibiotics registered in the point-prevalence surveys, Norway 2015–2023

CTX: cefotaxime; MDZ: metronidazole; SMX: sulfamethoxazole; TMP: trimethoprim.

^a^ Empirical intravenous first-line or alternative treatment recommended in national guidelines are marked with ^a^ in the figure (see Supplementary Table S1 for details in guidelines).

Major gentamicin combinations: (i) benzylpenicillin and gentamicin, (ii) ampicillin and gentamicin, (iii) ampicillin, gentamicin and metronidazole.

Urinary tract infections (UTI) include both upper and lower UTI.

In all patients treated for UTI, ampicillin and gentamicin were the fifth most frequently used UTI regimen, increasing from 3.5% in 2015 to 9% in 2023. Pivmecillinam or mecillinam was the most common UTI treatment, with use declining from 27.7% in 2015 to 21.8% in 2023. Use of trimethoprim/sulfamethoxazole (TMP/SMX) or trimethoprim increased from 15.0% in 2015 to 21.6% in 2023. Cephalosporins peaked at 21.0% in 2022 before declining to 17.5% in 2023 (Figure 5A). Information on the distribution of gentamicin use across departments with intensive care units (ICUs) is provided in Supplementary Table S7, while detailed annual numbers and proportions underlying Figure 5 are presented in Supplementary Table S8.

In patients with sepsis (Figure 5B), major gentamicin combinations (ampicillin and gentamicin, ampicillin, gentamicin and metronidazole, and benzylpenicillin and gentamicin), ranked as the third most frequently used antibiotic regimen, accounting for 12.2% throughout the study period. The use of cephalosporins, the most prevalent antibiotics for the treatment of sepsis, declined from 21.3% in 2015 to 19.0% in 2023. Supplementary Table S9 shows that the use of piperacillin–tazobactam for the treatment of sepsis reached the highest level, 18.6%, in 2021.

Among patients with LRTI (Figure 5C), benzylpenicillin and gentamicin were used in only 2.8% of patients. Benzylpenicillin or phenoxymethylpenicillin monotherapy was the most common regimen, accounting for more than 30.0% throughout the study period. Cephalosporins (mainly cefotaxime) were the second most frequently used class, as shown in Supplementary Table S10. In all patients with IAI, piperacillin–tazobactam was the most frequently used antibiotic (Figure 5D). Its use decreased from 60.6% in 2015 to ca 41% during 2017–2019, followed by a slight increase after 2019. The combination of ampicillin, gentamicin and metronidazole increased from 0% in 2015 to 13.3% in 2023 in patients with IAI. Further details on the annual use of individual IAI regimens are available in Supplementary Table S11.

## Discussion

During the study period, 26.9–28.1% of hospitalised patients received antibiotics, which is lower than the prevalence reported in European acute care hospitals in 2022 (35.1%) [18] and in Swedish hospitals (36.1%) [19]. In Norway, the preferred antibiotics in antibiotic stewardship programmes include β-lactamase-sensitive penicillins, extended-spectrum penicillins, β-lactamase-resistant penicillins, TMP/SMX and aminoglycosides [11], which coincide with the Access group in WHO AWaRe categories [20]. In our study, the β-lactamase-sensitive penicillins were the most frequently used antibiotics during the study period. This contrasts recent PPS data reported by the Public Health Agency of Sweden (PHAS) in 2023, showing that more than half (53.4%) of antibiotic courses involved broad-spectrum antibiotics, with piperacillin–tazobactam being the most frequently used [19].

The substantial use of β-lactamase-sensitive penicillins is consistent with other Norwegian reports [11] and the latest European Centre for Disease Prevention and Control (ECDC) report [18]. In 2021, the use of third-generation cephalosporins (14.1%) surpassed that of β-lactamase-sensitive penicillins (12.6%) in our study. It is unlikely that this shift reflects changes in AMR epidemiology in Norway, institutional factors, or changes in treatment guidelines during the study period. However, this might be related to the COVID-19 pandemic and changes in the hospitalised population, since the use of third-generation cephalosporins measured in defined daily doses (DDD) per 1,000 inhabitants per day remained stable between 2020 and 2024 [11]. Additionally, the Norwegian action plan on antimicrobial resistance expired in 2020 [21] and was not replaced by a new strategy until 2024, potentially creating a gap in national-level guidance on antibiotic stewardship measures [21].

Interestingly, the use of penicillins with β-lactamase inhibitors declined sharply from 2017 to 2019, due to periods of nationwide shortages of piperacillin-tazobactam [11]. The latest Norwegian data from sales statistics measured in DDD per 100 bed-days show an increase in the use of penicillins with β-lactamase inhibitors between 2020 and 2024 [11]. In European acute care hospitals, combinations of penicillins, including β-lactamase inhibitors, were the most frequently used agents in 2022–2023 [18]. Global trends in antibiotic consumption measured in DDD per 1,000 inhabitants per day also demonstrate increased use in broad-spectrum penicillins especially in high-income countries, during 2021–2023 [22]. Collectively, these findings underscore that antibiotic stewardship and surveillance must be integral to clinical care to ensure judicious antibiotic use.

Previous reports have shown that the COVID-19 pandemic affected antibiotic prescribing practices [13]. This aligns with our findings suggesting a shift in trends for Access and Watch/Reserve antibiotics after the onset of the pandemic, while the increasing pre-pandemic trend in gentamicin use was no longer observed.

Aminoglycosides were only the ninth most commonly prescribed antibiotic class in our study, despite gentamicin being an essential antibiotic in empirical treatment guidelines for severe infections in Norway [12]. This observation aligns with Plachouras et al., who found gentamicin to rank ninth in terms of total antimicrobial use across 28 European Union/European Economic Area countries in PPS [23]. However, aminoglycoside use in our study (5.0% at the prescription level in 2023) was higher than the 3.1% reported in the 2024 ECDC report [18]. The ECDC data also show substantial intercountry variation, with aminoglycoside use ranging from 0.6% in the Netherlands to 8.0% in Bulgaria and 12.8% in Kosovo^*^, reflecting major differences in practice and AMR rates across Europe. Notably, the highest relative use of aminoglycosides in European hospitals was observed in paediatric and ICU patients. The low use of aminoglycosides in ICU patients in our study is likely consistent with national guidelines, which discourage their use in septic shock, despite their role in the empirical treatment of severe infections, including sepsis [12].

In Norway, the recommendation for combination therapy with narrow-spectrum β-lactam antibiotics and gentamicin is supported by favourable national AMR epidemiology [11]. In many European countries, gentamicin also demonstrates a more favourable resistance profile than third-generation cephalosporins against common pathogens causing severe infections [10] and should be considered more frequently as a strategy to spare broader-spectrum antibiotics. Several factors may contribute to the relatively low use of aminoglycosides in European hospitals. First, concerns about side effects, such as nephrotoxicity and ototoxicity, may lead clinicians to avoid their use. However, recent studies indicate that there is no association between aminoglycoside use and worse renal outcomes [9,24]. Second, gentamicin is known for its cumbersome administration requirements, necessitating therapeutic monitoring. Interestingly, when considering the proportion of patients receiving antibiotics treated with gentamicin (patient-level), we observed an increase from 4.0% in 2015 to 5.8% in 2023. This trend aligns with the latest Norwegian antibiotic surveillance report, which indicates a low but increasing sales rate of aminoglycosides compared with other systemic antibiotics [11].

International and Norwegian guidelines recommend combination therapy with β-lactams and aminoglycosides for the empirical treatment of UTIs [12,25]. In our study, UTIs were the primary indication for gentamicin treatment. However, in 2023, almost twice as many patients received broad-spectrum cephalosporins as received ampicillin combined with gentamicin. Although cefotaxime is the recommended alternative when gentamicin is contraindicated (e.g. in kidney failure), this suggests a potential area for improvement in antibiotic stewardship programmes. The high proportion of patients who received ampicillin or amoxicillin monotherapy, TMP/SMX or pivmecillinam for UTIs may reflect step-down or targeted therapy as per guidelines.

The role of aminoglycosides in infections outside the urinary tract is controversial, and guidelines recommend their use only in combination therapy [26]. In Norway, gentamicin combinations are recommended as first-line treatment for LRTIs, IAIs and sepsis [12], supported by favourable AMR epidemiology [11]. To our knowledge, there are few other national or international guidelines in which aminoglycosides combined with narrow-spectrum antibiotics are part of first-line treatment for serious infections, although aminoglycosides are used in critically ill patients with sepsis [27]. The low use of gentamicin outside the urinary tract shown in our study, likely reflects the use of gentamicin combinations mainly as empirical therapy at admission.

The recommendation to use gentamicin for empirical combination therapy in LRTIs in the Norwegian guidelines is unusual and not widely endorsed in other guidelines, likely due to concerns regarding poor lung tissue penetration [28]. Benzylpenicillin monotherapy was the most frequently used treatment for LRTIs. However, the use of third-generation cephalosporins has markedly increased, highlighting the need for attention in antibiotic stewardship programmes.

In the treatment of IAIs, the Norwegian guidelines recommend a triple regimen of ampicillin, gentamicin and metronidazole, despite limited clinical trial evidence, reflecting a long-standing treatment tradition and the consideration that the triple regimen is less likely to promote AMR while maintaining adequate antimicrobial coverage. However, piperacillin-tazobactam was the most frequently used antibiotic. Its use markedly decreased over the past 5–7 years, partly due to periods of limited access [11]. Concurrently, there has been a substantial increase in the use of combination therapy with ampicillin, gentamicin and metronidazole, in line with the treatment guidelines, possibly reflecting a strategy to reduce reliance on piperacillin-tazobactam.

A key strength of this study is the use of nationwide data collected over a nine-year period, with consistent data collection during the same months each year. The inclusion of most Norwegian hospitals further enhances generalisability. Although our findings are relevant to antimicrobial stewardship policies, there are some limitations to the study. The results could not be adjusted for institutional factors. The lack of detailed information on hospital characteristics further constrains the interpretation of the findings. The Norwegian PPS protocol lacks information on the variable ‘*change of antibiotic agent*', which is included in the protocols of other European countries [18]. Consequently, we could not assess compliance with national guidelines due to missing data on empirical and targeted treatment, duration of treatment and microbiological findings.

Point-prevalence data from European initiatives [18] and the Global-PPS, an international surveillance programme designed to measure and monitor antimicrobial prescribing worldwide, can support national and international stewardship efforts in line with the WHO recommendations for establishing surveillance systems [1]. These goals align with the latest Norwegian national AMR strategy, which commits to global collaboration and the adoption of new technologies [21]. Importantly, although surveillance data alone are insufficient to support guideline changes, they provide valuable real-world information on current prescribing practices while awaiting evidence from ongoing randomised controlled trials, such as the Aminoglycosides in Early Sepsis (AGES) trial (NCT06712641), to inform future recommendations.

The Norwegian PPS is conducted up to four times annually, whereas the ECDC PPS is performed every 5 years [18]. The Global-PPS includes assessments of guideline adherence and microbiological sampling, enabling international benchmarking [29], which Norway currently lacks. Given that more than a decade has passed since the Norwegian PPS was introduced, revision is warranted to improve comparability with European and global systems.

## Conclusion

Antibiotic use in Norwegian hospitals remained stable and lower than that reported elsewhere in Europe, but a post-pandemic shift towards broader-spectrum antibiotics suggests a drift from stewardship goals. Gentamicin use remained modest despite guideline support, indicating the potential to increase narrow-spectrum combination therapy. Strengthened stewardship efforts are needed, and PPS represent an important tool for monitoring and evaluating antibiotic use.

## Data Availability

Data are available from the Norwegian Institute of Public Health in accordance with national surveillance regulations. All data presented in the manuscript or supplementary material are available from the corresponding author upon reasonable request.

## *Note

This designation is without prejudice to positions on status and is in line with UNSCR 1244/99 and the IСJ Opinion on the Kosovo declaration of independence.

## Supplementary Data

796000 Supplementary Material
