# Supplementary material for: Gentamicin and antibiotic use in Norwegian hospitals: a study from 35 point-prevalence surveys, 2015 to 2023
Source: Euro Surveill. 2026 Oct 22;31(42):2500969. doi: 10.2807/1560-7917.ES.2026.31.42.2500969 (PMC13695006; doi:10.2807/1560-7917.ES.2026.31.42.2500969)
Supplement: Supplementary file 1 [file 25-00969_TONBY_Supplement.pdf]

SUPPLEMENTARY MATERIAL

This supplementary material is hosted by Eurosurveillance as supporting information alongside the article "Gentamicin and antibiotic use in Norwegian hospitals: a study from 35 point-prevalence surveys, 2015 to 2023", on behalf of the authors, who remain responsible for the accuracy and appropriateness of the content. The same standards for ethics, copyright, attributions and permissions as for the article apply. Supplements are not edited by Eurosurveillance and the journal is not responsible for the maintenance of any links or email addresses provided therein.

Description of content.

Supplementary Methods and Data Collection Template (page 1-5). Additional information about data collection, including translated version of the registration template and ward registration form.

Supplementary Table S1 (page 6). Excerpt from current national guideline for empirical antibiotic use in hospitals.

Supplementary Table S2 (page 7-8). Antibiotic class and substance according to WHOs Access, Watch/Reserve categories underlying Figure 3.

Supplementary Figure S1A and S1B (page 9). Number of participating hospitals and patients receiving antibiotics per survey.

Supplementary Table S3 (page 10). The percentage of patients on different gentamicin combinations underlying Figure 4.

Supplementary Table S4 (page 11). Frequency distribution for each ATC-group with substances, 2015-2023.

Supplementary Table S5 (page 12-13). Antibiotic prescriptions, 2015–2023 underlying Figure 2.

Supplementary Table S6 (page 14). Pre-pandemic trend, level change and post-pandemic slope change of modelled gentamicin use and overall antibiotic use (Access and Watch/Reserve) in interrupted time series analyses.

Supplementary Table S7 (page 14). Distribution of total gentamicin use across ICU departments.

Supplementary Figure S2 (page 15) Distribution of treatment indications among gentamicin treated patients.

Supplementary Table S8 -11 (page 16-19) Distribution of major antibiotic regimens in urinary tract infections, sepsis, lower respiratory tract infections and intra-abdominal infections underlying Figure 5.

Supplementary Methods - Study design and data collection

Data were collected using a standardized data collection protocol developed by the Norwegian Institute of Public Health (NIPH). Minor changes were made to the protocol in 2019, including specification on how indications should be registered and regarding medical prophylaxis. Apart from these changes data collection was consistent throughout the study period. The data set from NIPH included information on the year, data and month of the data collection, the total number of patients admitted on the day of PPS, and the total number of patients on antibiotics at each department. For each individual in the dataset, the following variables were registered: age (≥20 years), sex (male/female), department specialty, suspected origin of infection, type of indication (registered at the day of PPS) and if the treatment indication was documented in the patient records. The following antibiotics according to ATC-index (ATCDDD - ATC/DDD Index) were registered: (J01 antibiotics for systemic use), (A07A A09 vancomycin) and (P01A B01 metronidazole) (Supplementary Table S1). The age cut-off at ≥20 years is due to the designated age groups in the Norwegian protocol.

Classified as ECDC NORMAL

DATA COLLECTION TEMPLATE (Excerpt from registration template concerning antibiotic variables from NOIS-PIAH, Registreringsmal for sykehus - FHI) *Translated into English with assistance from ChatGPT version 1.2035.336. The translation has been quality assured.*

Antibiotic variables

Number of patients treated with antibiotics

The number of admitted patients who receive at least one of the following anti-infective agents systemically (referred to as antibiotics in the rest of the registration template) must be recorded:

All types of antibiotics in the following ATC classes:

- J01 Antibacterials for systemic use (incl. the urinary antiseptic methenamine (Hiprex))
- J02 Antimycotics for systemic use

The following individual antibiotics listed under other ATC classes:

- A07 AA09 Vancomycin – treatment of *C. difficile*, listed under intestinal anti-infectives
- A07 AA12 Fidaxomicin – treatment of *C. difficile*, listed under intestinal anti-infectives
- P01 AB01 Metronidazole – for anaerobic infections, listed under antiparasitic products
- J04 AB02 Rifampicin – antistaphylococcal agent, listed under antimycobacterial agents

If none of the patients receive antibiotics, zero (0) must be recorded. Clarifications:

- All systemic use of antibiotics in the list must be recorded, except when used for the treatment of tuberculosis.
- The tuberculosis medicines in the list must only be recorded when they are used to treat disease caused by bacteria other than *Mycobacterium tuberculosis*.
- Locally acting antibiotics (creams, eye drops, vaginal pessaries and similar) must not be recorded.

Antibiotic variables to be recorded per patient

Sex - The patient's sex, as indicated by the third individual digit of the personal identification number, must be recorded: Female / Male / Unknown.

Age- The patient's age must be registered in age groups (years): 0–4 / 5–9 / 10–19 / 20–29 / 30–39 / 40–49 / 50–59 / 60–69 / 70–79 / 80+ / Unknown.

Product name- The product name must be stated, e.g. Ery-Max.

Dose- The dose per administration (single dose) must be stated: integer/decimal number.

Dose unit - The unit of measurement must be stated: Gram (g) / Milligram (mg) / Million international units (million IU) / Millilitre (ml) / Tablet (tab). The number of millilitres or tablets per dose should only be stated for combination products, e.g. Bactrim.

Number of administrations per day- The number of administrations per day must be recorded as a whole number between 1 and 8. If an antibiotic is discontinued during the survey day, the number of administrations per day in the course should be recorded, even if the patient only received the morning dose on the survey day. Antibiotics that are not given every day should only be recorded if they are given on the survey day. In that case, dose and number of administrations per day should be recorded as if they were given every day.

Route of administration- Oral (O) / Intramuscular (IM) / Intravenous (IV) / Inhalation (Inhal) / Rectal (R) / Unknown.

Type of indication All use of antibiotics in the list above must be recorded and classified. This also applies when a Healthcare-associated infection HAI does not meet the criteria of one of the case definitions. When registering antibiotics, it is sufficient to record what the physicians believe they are treating (intention).

Type of indication must indicate whether the antibiotic is given for prevention or treatment of an infection, and whether the prescription concerns treatment of a community-acquired or healthcare-associated infection, or whether the antibiotic given for prevention is medical or surgical prophylaxis. One of the following codes (given in parentheses) must be used:

Treatment intention: treatment of community-acquired infection (CAI)/healthcare-associated infection (HAI).

Surgical prophylaxis: given as a single dose (Surpro1) / given several times within one day (Surpro2) / given for more than one day (Surpro3). Surpro1 or Surpro2 must have been given within 24 hours preceding the survey time (08:00 on the survey day). If, in addition, a patient has received surgical prophylaxis two days before or on the survey day, the prophylaxis must be recorded as Surpro3.

Classified as ECDC NORMAL

Medical prophylaxis (Medpro) must be recorded when antibiotics are given to prevent an infection, but not in relation to a surgical procedure. When medical prophylaxis is registered, there must be no suspicion of, and no documented, ongoing infection.

Other than prevention/treatment of infection, e.g. erythromycin used for prokinetic treatment (Other). Unknown classification of antibiotic treatment (Unknown).

Indication for antibiotic use
One of the following indications for antibiotic use must be selected:

- Upper urinary tract infection
- Lower urinary tract infection
- Asymptomatic bacteriuria
- Infection of the eye, ear, nose, throat or mouth
- Lower respiratory tract infection
- Skin and soft tissue infection, excl. postoperative wound infection
- Surgical site infection, incl. postoperative wound infection and organ/space infection
- Bone and joint infection
- Central nervous system infection
- Obstetric or gynaecological infection, incl. sexually transmitted infection in women
- Prostatitis, epididymitis, incl. sexually transmitted infection in men
- Neutropenic fever
- Cardiovascular system infection
- Gastroenteritis, excl. antibiotic-associated diarrhoea
- Antibiotic-associated diarrhoea
- Intra-abdominal infection, excl. postoperative intra-abdominal infection
- Laboratory-confirmed bloodstream infection
- Clinical sepsis with presumed pulmonary focus
- Clinical sepsis with presumed urinary focus
- Clinical sepsis with presumed abdominal focus
- Clinical sepsis with another presumed focus
- Clinical sepsis with uncertain focus
- Other / infection with uncertain focus without clinical sepsis
- Unknown

Clarifications:

- The indication should be surgical site infection (incl. postoperative wound infection and organ/space infection) when the classification is surgical prophylaxis (1, 2 or 3).
- The focus that the prophylaxis is directed against must be recorded as the indication when the type of indication is medical prophylaxis. For example, the indication should be lower urinary tract infection and the type of indication is medical prophylaxis when using methenamine (Hiprex).
- When antibiotics are given as medical prophylaxis and several types of infections are possible outcomes, the most serious outcome should be recorded as the indication.
- The most serious indication should also be recorded when several infections with different foci are being treated with the same antibiotic.
- If one patient receives several systemic antibiotics for the same indication, all drugs must be recorded separately, and the same indication and classification must be given for each drug.
- Is the indication documented in writing? Is the indication documented in writing in the medical record or drug chart? Yes/No/Unknown. If information on this is not available, "Unknown" must be recorded. If, after reviewing charts etc., no documentation of the indication is found, "No" must be recorded.
- Was a microbiological sample taken? Was a relevant microbiological sample taken before or at the start of antibiotic treatment? Yes/No/Unknown.
- Urine dipsticks are not considered a microbiological sample. The test result does not have to be available at the time of the survey. If information about sampling is not available, "Unknown" must be recorded.

Quality assurance
At a minimum, the following should be checked:

- All patients admitted at 08:00 a.m. are included in the survey.

Classified as ECDC NORMAL

- All infections recorded as healthcare-associated meet the criteria in the definition (not shown here).
- All registered HAIs meet the criteria in the relevant case definition. Check laboratory reports and documented clinical information. If there are very many infections, spot checks may be performed.
- Age and sex are specified for all patients who received antibiotics, as well as which antibiotics they received (product name), dosing, administration, type of indication and indication, whether the indication is documented in writing and a microbiological sample was taken.
- That there are no logical inconsistencies in the recording of antibiotic use. For example, prophylaxis is stated against a type of infection for which antibiotics are normally not given to prevent infection, or that the dose is not consistent with the unit of measurement (not 3000 g, but 3 g or 3000 mg)

Classified as ECDC NORMAL

Systemic antibiotic use – ward registration form

Clarifications:

- Do not record data from multiple wards on the same form
- If a patient is treated with more than one systemic antibiotic (for one or several indications), each antibiotic must be recorded separately (one line per antibiotic).
- If a patient is treated with more than one systemic antibiotic for the same indication, the indication should be recorded multiple times.
-

Registered by:____

Ward name: ____

Date: ____

Number of admitted patients at 08:00 a.m.: ____

Department speciality:

Number of patients on antibiotics: ____

**Patient- id (internal use only)**	**Sex**	**Age**	**Product name (antibiotic)**	**Dose per administration**	**Dose Unit**	**Number of administrations per day**	**Route of administration**	**Type of indication**	**Indication**	**Is indication documented in the patient records?**	**Micro-biological sampling**

**Variable**	**Comment**
Product name (antibiotic)	e.g. Ery-Max
Dose Unit	Gram (g) / Milligram (mg) / Million international units (million IU) / Millilitre (ml)[1] / Tablet (tabl.)[1] [1] *Combination products only (e.g. Bactrim)*
Route of admiration	Oral (O) / Intramuscular (IM) / Intravenous (IV) / Inhalation (Inhal) / Rectal (R) /Unknown
Type of indication	Community-acquired infection (CAI) / Healthcare-associated infection (HAI) / Surpro1 - prophylaxis given as single dose) / Surpro2 - prophylaxis given multiple times during 24h / Surpro3 - prophylaxis given more than 24h / Medical prophylaxis (Medpro) / Other / Unknown
Indication	The type of infection targeted by the treatment or prevented by the prophylaxis, or unknown; see the indication list in the template.
Is indication documented in the patient records?	Yes / No / Unknown
Is microbiological sample taken?	Yes / No / Unknown

See template for full description of variables

Classified as ECDC NORMAL

Supplementary Table S1: Excerpt from current national guideline for empirical antibiotic use in hospitals (assessed May 2026)

Infection Category	Clinical Situation / Focus	Recommended Treatment	Alternative treatment 1	Alternative treatment 2
**Sepsis (without shock)**	Unknown focus	Benzylpenicillin IV 2.4 g × 6 + Gentamicin* IV 6-7 mg/kg × 1	Cefotaxime IV 2 g × 3	Piperacillin/Tazobactam IV 4/0.5 g × 4
	Lower respiratory tract (Community-acquired)	Benzylpenicillin IV 3 g × 4 + Gentamicin* IV 6 mg/kg × 1	See section "Lower Respiratory Tract Infections"	See section "Lower Respiratory Tract Infections"
	Lower respiratory tract (Hospital-acquired)	Piperacillin/Tazobactam IV 4/0.5 g × 4	See section "Lower Respiratory Tract Infections"	See section "Lower Respiratory Tract Infections"
	Urinary tract	Ampicillin IV 2 g × 4 + Gentamicin* IV 6–7 mg/kg × 1	Cefotaxime IV 2 g × 3	
	Abdomen	Ampicillin IV 2 g × 4 + Gentamicin* IV 6–7 mg/kg × 1 + Metronidazole IV 1.5 g loading dose, then 1 g × 1	Piperacillin/Tazobactam IV 4/0.5 g × 4	Cefotaxime IV 2 g × 3 + Metronidazole IV 1.5 g loading dose, then 1 g x 1
	Skin/soft tissue	Cloxacillin IV 2 g × 6 + Gentamicin* IV 6 mg/kg × 1	Cefotaxime IV 2 g × 3	Piperacillin/Tazobactam IV 4/0.5 g × 4
**Septic shock**	Any focus	Piperacillin/Tazobactam IV 4/0.5 g × 4 (Loading dose over 30 min, then over 3 h, repeat immediately after loading dose)		
**Urinary Tract Infections**	Pyelonephritis	Ampicillin IV 1 g × 4 + Gentamicin* IV 6 mg/kg × 1	Cefuroxime IV 750 mg x 3	Cefotaxime IV 1 g × 3
**Abdominal Infections**	Appendicitis, cholecystitis, cholangitis, diverticulitis, secondary peritonitis	Ampicillin IV 2 g × 4 + Gentamicin* IV 6 mg/kg × 1 + Metronidazole IV 1.5 g loading dose, then 1 g × 1	Cefotaxime IV 1 g x 3 OR Ceftriaxone IV 2g x 1 + Metronidazole IV 1.5 g x 1 loading dose, then 1 g x 1	Piperacillin/Tazobactam IV 4/0.5 g x 3
**Lower Respiratory Tract Infections**	Pneumonia, community-acquired (mild/moderate, CRB65 0–2, unknown agent)	Benzylpenicillin IV 1.2 g × 4		
	Pneumonia, community-acquired (severe, CRB65 3–4, unknown agent)	Benzylpenicillin IV 3 g × 4 + Gentamicin* IV 6 mg/kg × 1	Cefotaxime IV 2 g x 3	
	Pneumonia, community-acquired (very severe, CRB65 3–4)	Cefotaxime IV 2 g × 3 + Ciprofloxacin IV 400 mg × 3 OR Azitromycin IV 500 mg x 1	Piperacillin/Tazobactam IV 4/0.5 g x 4 + Ciprofloxacin IV 400 mg × 3 OR Azitromycin IV 500 mg x 1	
	Pneumonia, hospital-acquired (uncomplicated)	Benzylpenicillin IV 1.2 g × 4 + Gentamicin* IV 6 mg/kg × 1	Cefotaxime IV 1 g x 3	
	Pneumonia, hospital-acquired (complicated)	Piperacillin/Tazobactam IV 4/0.5 g × 4		
	Infectious COPD exacerbation	Ampicillin IV 1 g × 4	Cefotaxime IV 1 g x 3	
	Aspiration pneumonia	Benzylpenicillin IV 1.2 g × 4	Cefotaxime IV 2 g x 3	Piperacillin/Tazobactam IV 4/0.5 g x 4

**Caution if severe chronic kidney disease (GFR <30ml/min), high age with comorbidity and polypharmacy, nephrotoxic medication or organ transplant recipient, hypovolemia or circulatory failure.*

The full version of the guidelines is available at The Norwegian Directorate of Health; Antibiotic therapy in hospitals: Antibiotika i sykehus - Helsedirektoratet. The national guideline was first published in 2013 and subsequently revised for respiratory tract infections (September 2020), urinary tract infections (March 2021), abdominal infections (March 2022), and sepsis (August 2022). Throughout the study period (2015–2023), gentamicin in combination with a narrow-spectrum beta-lactam remained the first-line empirical treatment for sepsis without shock (unknown origin, community-acquired lower respiratory tract infections, urinary tract infections, and primary abdominal infections). In December 2021, the threshold for avoiding gentamicin in empirical regimens was lowered from eGFR <40 ml/min to <30 ml/min, and in April 2023 the chapter on gentamicin concentration monitoring was updated.

Classified as ECDC NORMAL

Supplementary Table S2: Antibiotic class and substance prescribed in study population according to WHO Access, Watch/Reserve categories, underlying Figure 3

Class	Antibiotic	Access	Watch/Reserve
**Aminoglycosides**	Amikacin	26	0
**Penicillins**	Amoxicillin	2,561	0
**Beta-lactam/beta-lactamase-inhibitor**	Amoxicillin/clavulanic-acid	227	0
**Penicillins**	Ampicillin	3,792	0
**Macrolides**	Azithromycin	0	198
**Monobactams**	Aztreonam	0	35
**Penicillins**	Benzylpenicillin	9,419	0
**First-generation-cephalosporins**	Cefalexin	260	0
**First-generation-cephalosporins**	Cefalotin	2,909	0
**First-generation-cephalosporins**	Cefazolin	3,188	0
**Fourth generation-cephalosporins**	Cefepime	0	1
**Other-cephalosporins**	Cefiderocol	0	1
**Third generation-cephalosporins**	Cefixime	0	7
**Third generation-cephalosporins**	Cefotaxime	0	8,731
**Fifth-generation cephalosporins**	Ceftaroline-fosamil	0	3
**Third generation-cephalosporins**	Ceftazidime	0	342
**Third generation-cephalosporins**	Ceftazidime/avibactam	0	10
**Fifth-generation cephalosporins**	Ceftolozane/tazobactam	0	7
**Third generation-cephalosporins**	Ceftriaxone	0	1,225
**Second-generation-cephalosporins**	Cefuroxime	0	1,470
**Fluoroquinolones**	Ciprofloxacin	0	2,892
**Glycopeptides**	Dalbavancin	0	8
**Daptomycin**	Daptomycin	0	31
**Penicillins**	Dicloxacillin	1,931	0
**Tetracyclines**	Doxycycline	1,640	0
**Carbapenems**	Doripenem	0	1
**Carbapenems**	Ertapenem	0	112
**Macrolides**	Erythromycin	0	512
**Penicillins**	Phenoxymethylpenicillin	1,337	0
**Penicillins**	Flucloxacillin	2	0
**Phosphonics**	Fosfomycin	0	4
**Aminoglycosides**	Gentamicin	3,705	0
**Carbapenems**	Imipenem/cilastatin	0	165
**Macrolides**	Clarithromycin	0	67
**Lincosamides**	Clindamycin	3,028	0
**Penicillins**	Cloxacillin	4,461	0
**Amphenicols**	Chloramphenicol	14	0
**Polymyxins**	Colistin	0	6
**Fluoroquinolones**	Levofloxacin	0	57
**Oxazolidinones**	Linezolid	0	345
**Penicillins**	Mecillinam	223	0

Classified as ECDC NORMAL

**Carbapenems**	Meropenem	0	1,893
**Tetracyclines**	Metacycline	0	1
**Imidazoles**	Metronidazole	4,002	0
**Fluoroquinolones**	Moxifloxacin	0	22
**Fluoroquinolones**	Ofloxacin	0	3
**Beta-lactam/beta-lactamase-inhibitor**	Piperacillin/tazobactam	0	8,143
**Penicillins**	Pivmecillinam	2,108	0
**Streptogramins**	Pristinamycin	0	1
**Macrolides**	Spiramycin	0	1
**Tedizolid**	Tedizolid	0	20
**Glycopeptides**	Teicoplanin	0	5
**Glycylcyclines**	Tigecycline	0	4
**Aminoglycosides**	Tobramycin	0	166
**Trimethoprim-derivatives**	Trimethoprim	524	0
**Sulfonamide-trimethoprim-combinations**	Sulfamethoxazole/trimethoprim	5,042	0
**Glycopeptides**	Vancomycin	0	1,468
**Total**		**50,399**	**27,957**

Blue colour represents antibiotics in Access category.** Watch and Reserve categories were combined due to low levels of Reserve antibiotics. Classification follows the WHO AWaRe framework [The WHO AWaRe (Access, Watch, Reserve) antibiotic book, Geneva: Word Health Organization; 2022].*

Classified as ECDC NORMAL

Supplementary Figure S1A and S1B

(A) Number of participating hospitals per survey

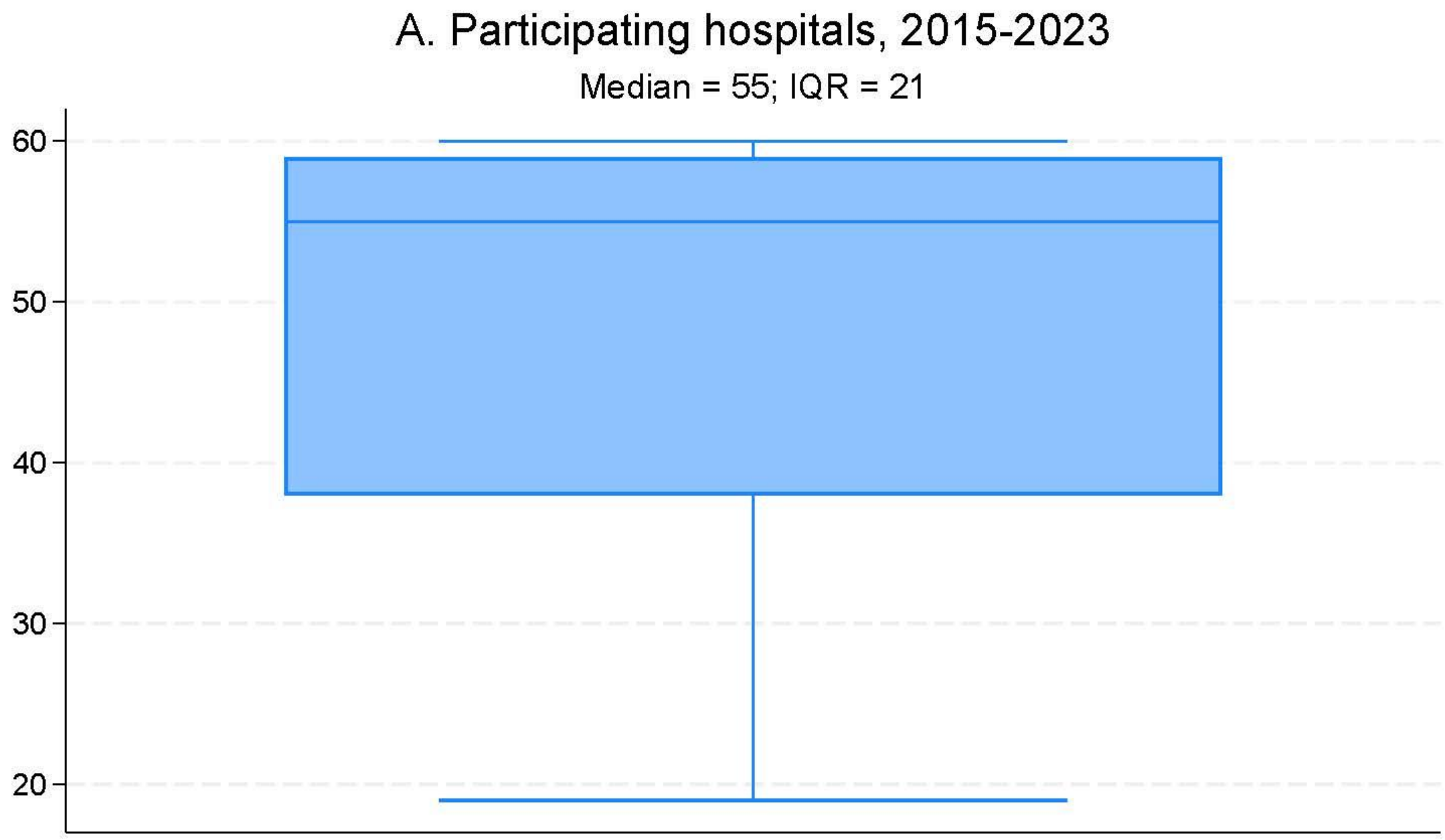

(B) Number of patients receiving antibiotics per survey

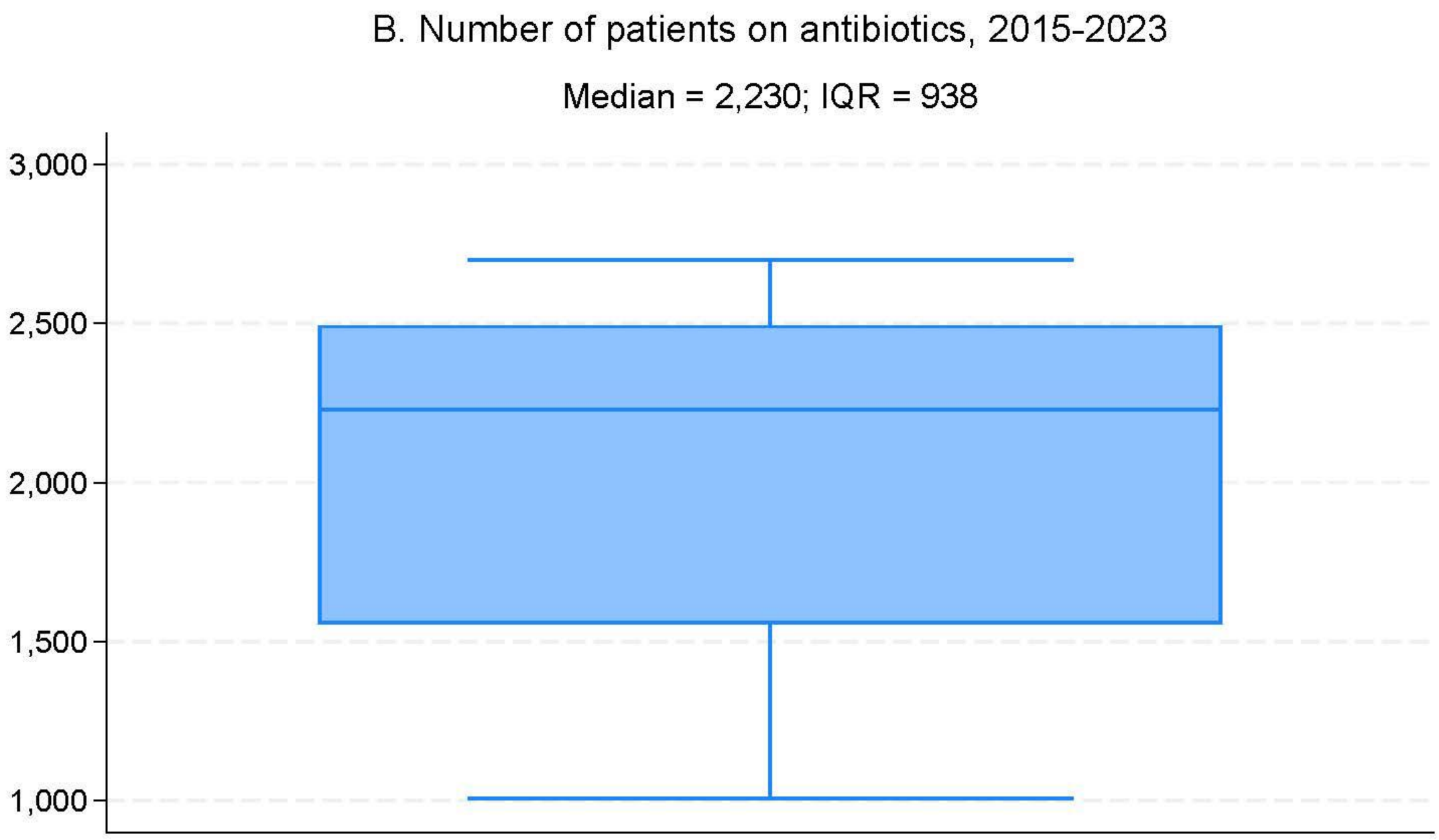

Classified as ECDC NORMAL

Supplementary Table S3: The percentage of patients on different gentamicin combinations during the study period, overall gentamicin use and prevalence of patients on antibiotics 2015-2023, underlying Figure 4.

	2015		2016		2017		2018		2019		2020		2021		2022		2023	
**Gentamicin regimens**	**N**	**%**	**N**	**%**	**N**	**%**	**N**	**%**	**N**	**%**	**N**	**%**	**N**	**%**	**N**	**%**	**N**	**%**
**Benzylpenicillin and Gentamicin**	79/ 5251	1.5	131/ 7512	1.7	147/ 7989	1.8	178/ 8362	2.1	196/ 8397	2.3	105/ 6349	1.7	112/ 7001	1.6	108/ 7998	1.4	114/ 7727	1.5
**Ampicillin and Gentamicin**	53/ 5251	1.0	90/ 7512	1.2	120/ 7989	1.5	170/ 8362	2.0	140/ 8397	1.7	136/ 6349	2.1	158/ 7001	2.3	162/ 7998	2.0	160/ 7727	2.1
**Other Gentamicin combinations***	49/ 5251	0.9	83/ 7512	1.1	74/ 7989	0.9	64/ 8362	0.8	53/ 8397	0.6	54/ 6349	0.9	42/ 7001	0.6	58/ 7998	0.7	29/ 7727	0.4
**Ampicillin, Metronidazole and Gentamicin**	2/ 5251	0.0	4/ 7512	0.1	24/ 7989	0.3	60/ 8362	0.7	84/ 8397	1.0	58/ 6349	0.9	59/ 7001	0.8	91/ 7998	1.1	109/ 7727	1.4
**Gentamicin monotherapy**	12/ 5251	0.2	12/ 7512	0.2	32/ 7989	0.4	27/ 8362	0.3	30/ 8397	0.4	27/ 6349	0.4	19/ 7001	0.3	15/ 7998	0.2	16/ 7727	0.2
**Cloxacillin and Gentamicin**	15/ 5251	0.3	11/ 7512	0.1	21/ 7989	0.3	20/ 8362	0.2	21/ 8397	0.3	14/ 6349	0.2	18/ 7001	0.3	22/ 7998	0.3	17/ 7727	0.2
**Overall gentamicin use**	210/ 5251	4.0	331/ 7512	4.4	418/ 7989	5.2	519/ 8362	6.2	524/ 8397	6.2	394/ 6349	6.2	408/ 7001	5.8	456/ 7998	5.7	445/ 7727	5.8
**Prevalence** of patients on antibiotics**	5251/ 19101	27.5	7512/ 27939	26.9	7989/29057	27.5	8362/ 30541	27.4	8397/ 29902	28.1	6349/ 23151	27.4	7001/ 25528	27.4	7998/ 28546	28.0	7727/ 27781	27.8

** Other Gentamicin combinations include combination of gentamicin with one of the following substances: Clindamycin, Metronidazole, Cefalotin, Amoxicillin, Ciprofloxacin, Cefotaxime, Vancomycin, Piperacillin/tazobactam. These combinations may not be in accordance with national guidelines but reflect prescribing as recorded in the point prevalence survey and may represent targeted therapy** The denominator is equal to the number of admitted patients on the day of PPS each year*

Classified as ECDC NORMAL

Supplementary Table S4. Frequency distribution for each ATC-group with substances, 2015-2023

ATC-groups and substances in each group	Frequency
**J01 CE PCN beta-lactamase sensitive**<br>Benzylpenicillin (87.6 %) (9419/10756), phenoxymethylpenicillin	10,756
**J01D D and J01D I 3rd gen cephalosporins and higher generations**<br>Cefotaxime (84.5%) (8731/10327), ceftazidime, ceftriaxone (11.9 %) (1225/10327), ceftazidime – avibactam ceftalozan with beta-lactamase inhibitor	10,327
**J01C A PCN with extended spectrum**<br>Ampicillin (43.7 %) (3792/8684), amoxicillin (29.4 %) (2561/8684), mecillinam, pivmecillinam	8,684
**J01C R PCN β-lactamase inhibitors**<br>Piperacillin-tazobactam (97.3 %) (8143/8370), amoxicillin-clavulanate	8,370
**J01D B 1st gen cephalosporins**<br>Cefalexin, cefalotin, cefazolin	6,357
**J01C F PCN beta-lactamase resistant**<br>Cloxacillin, flucloxacillin, dicloxacillin	6,394
**J01 EA and J01E E Trimethoprim and sulfonamides**<br>Trimethoprim, trimethoprim-sulfamethoxazole	5,566
**J01X D01 Imidazole derivatives and P01A B Nitroimidazole derivatives**<br>Metronidazole	4,002
**J01G B03 Aminoglycosides**<br>Gentamicin (95 %) (3705/3897), tobramycin (4.3%) (166/3897) amikacin (0.7%) (26/3897)	3,897
**J01F F01 Lincosamides**<br>Clindamycin	3,028
**J01M A02 Fluoroquinolones**<br>Ciprofloxacin, levofloxacin, moxifloxacin, ofloxacin	2,974
**J01D F Carbapenems**<br>Meropenem, imipenem, ertapenem	2,171
**01A A Tetracyclines**<br>Doxycycline, tigecycline, metacyclin	1,645
**J01D C02 2nd gen cephalosporins**<br>Cefuroxime	1,470
**J01X A Glycopeptide antibacterials A07A A09 intestinal antiinfectives**<br>Vancomycin	1,468
**J01F A01 Macrolides**<br>Erythromycin, clatrithromycin, azithromycin, spiramycin	778
**J01D H Monobactams/J01X X, J01X A, J01B A, J01X B01, J01F G01** Others aztreonam, linezolid, tedizolid, teicoplanin, amphenicols, dalbavacin, daptomycin, fosfomycin, colistin, pristinamycin	469
**Total**	**78,356**

Abbreviations. PCN: penicillins.

Classified as ECDC NORMAL

Supplementary Table S5: Antibiotic prescriptions, 2015–2023 (n = 78,356), underlying Figure 2

	2015		2016		2017		2018		2019		2020		2021		2022		2023	
**Antibiotic classes**	**N**	**%**	**N**	**%**	**N**	**%**	**N**	**%**	**N**	**%**	**N**	**%**	**N**	**%**	**N**	**%**	**N**	**%**
**J01 CE** PCN β-lactamase sensitive	845/6150	13.7	1236/8689	14.2	1392/9378	14.8	1470/9915	14.8	1497/9969	15.0	1006/7506	13.4	1028/8184	12.6	1110/9483	11.7	1172/9082	12.9
**J01D D and J01D I** 3rd Gen Cephs and higher gen	794/6150	12.9	1104/8689	12.7	1249/9378	13.3	1218/9915	12.3	1289/9969	13.0	960/7506	12.8	1150/8184	14.1	1337/9483	14.1	1217/9082	13.4
**J01C A** PCN with extended spectrum	691/6150	11.2	911/8689	10.5	1014/9378	10.8	1081/9915	10.9	1137/9969	11.4	853/7506	11.4	901/8184	11.0	1034/9483	10.9	1062/9082	11.7
**J01C R** PCN with β-lactamase inhibitors	745/6150	12.1	1146/8689	13.2	865/9378	9.2	911/9915	9.2	865/9969	8.7	785/7506	10.5	966/8184	11.8	1099/9483	11.6	988/9082	10.9
**J01D B** 1st Gen Cephs	408/6150	6.6	598/8689	6.9	638/9378	6.8	804/9915	8.1	801/9969	8.0	699/7506	9.3	757/8184	9.2	796/9483	8.4	856/9082	9.4
**J01C F** PCN β-lactamase resistant	454/6150	7.4	654/8689	7.5	753/9378	8.0	836/9915	8.4	762/9969	7.6	600/7506	8.0	698/8184	8.5	876/9483	9.2	761/9082	8.4
**J01 EA and J01E E** Trimethoprim and sulfonamides	335/6150	5.4	563/8689	6.5	656/9378	7.0	730/9915	7.4	714/9969	7.2	570/7506	7.6	630/8184	7.7	702/9483	7.4	666/9082	7.3
**J01X D01** Metronidazole	292/6150	4.7	336/8689	3.9	492/9378	5.2	539/9915	5.4	592/9969	5.9	400/7506	5.3	355/8184	4.3	506/9483	5.3	490/9082	5.4
**J01G B03** Aminoglycosides	255/6150	4.1	362/8689	4.2	446/9378	4.8	545/9915	5.5	532/9969	5.3	408/7506	5.4	420/8184	5.1	473/9483	5.0	456/9082	5.0
**J01F F01** Lincosamides	277/6150	4.5	361/8689	4.2	414/9378	4.4	369/9915	3.7	388/9969	3.9	281/7506	3.7	272/8184	3.3	341/9483	3.6	325/9082	3.6
**J01M A02** Fluoroquinolones	309/6150	5.0	445/8689	5.1	430/9378	4.6	395/9915	4.0	343/9969	3.4	255/7506	3.4	249/8184	3.0	306//9483	3.2	242/9082	2.7

Classified as ECDC NORMAL

	2015		2016		2017		2018		2019		2020		2021		2022		2023	
**Antibiotic classes**	**N**	**%**	**N**	**%**	**N**	**%**	**N**	**%**	**N**	**%**	**N**	**%**	**N**	**%**	**N**	**%**	**N**	**%**
**J01D F** Carbapenems	193/6150	3.1	284/8689	3.3	251/9378	2.7	248/9915	2.5	253/9969	2.5	202/7506	2.7	239/8184	2.9	277/9483	2.9	224/9082	2.5
**01A A** Tetracyclines	106/6150	1.7	134/8689	1.5	177/9378	1.9	209/9915	2.1	257/9969	2.6	175/7506	2.3	157/8184	1.9	222/9483	2.3	208/9082	2.3
**J01D C02** 2nd Gen Cephs	218/6150	3.5	234/8689	2.7	243/9378	2.6	189/9915	1.9	186/9969	1.9	122/7506	1.6	105/8184	1.3	87/9483	0.9	86/9082	0.9
**J01X and A07A A09** Vancomycin	121/6150	2.0	193/8689	2.2	184/9378	2.0	185/9915	1.9	162/9969	1.6	110/7506	1.5	141/8184	1.7	182/9483	1.9	190/9082	2.1
**J01F A01** Macrolides	83/6150	1.3	88/8689	1.0	121/9378	1.3	127/9915	1.3	113/9969	1.1	53/7506	0.7	62/8184	0.8	74/9483	0.8	57/9082	0.6
**J01D H /J01X X, J01X A, J01B A, J01X B01, J01F G01** Others*	24/6150	0.4	40/8689	0.5	53/9378	0.6	59/9915	0.6	69/9969	0.7	23/7506	0.4	54/8184	0.7	61/9483	0.6	82/9082	0.9

Abbreviations. PNC: penicillins.
**Others: Linezolid, Aztreonam, Daptomycin, Tedizolid, Chloramphenicol, Dalbavancin, Colistin, Teicoplanin, Fosfomycin, Pristinamycin.*

Classified as ECDC NORMAL

Supplementary Table S6: Pre-pandemic trend, level change and post-pandemic slope change of modelled Gentamicin use and overall antibiotic use (Access and Watch/Reserve) in interrupted time series analyses, Norway 2015-2023 (n = 78,356).

Category		Coefficient	p-value	95 % confidence interval
**Gentamicin**	**Trend before the pandemic β_1**	0.105	0.001	0.066 to 0.143
	**Level change β_2**	-0.657	0.105	-1.451 to 0.138
	**Slope change β_3**	-0.112	0.013	-0.201 to -0.023
**Access***	**Trend before the pandemic β_1**	0.632	0.001	0.519 to 0.746
	**Level change β_2**	-5.920	0.001	-7.774 to-4.067
	**Slope change β_3**	-0.533	0.001	-0.725 to-0.340
**Watch/Reserve**	**Trend before the pandemic β_1**	-0.632	0.001	-0.746 to -0.519
	**Level change β_2**	5.920	0.001	4.067 to 7.774
	**Slope change β_3**	0.533	0.001	0.340 to 0.725

*The interrupted time series was modelled with ITSA in STATA. Visual inspection indicated that the pre-COVID-19 segment followed an approximately linear trend, supporting a linear pre-trend assumption. Homoskedasticity was assessed using residual plots, and normality of residuals using histograms and Q-Q-plots. Portmanteau/Ljung–Box test and autocorrelation function plots did not indicate low-order autocorrelation. We therefore fitted a linear interrupted time series regression with Newey–West standard errors, specifying lag (0). *Gentamicin is included in the Access category.*

Supplementary Table S7: Distribution of total gentamicin use across ICU department in PPS, Norway, 2015-2023.

ICU subdivision in PPS survey	Other antibiotics	Gentamicin	Total
**Surgical Intensive Care Unit**	3,083	81	3,164
**Medical Intensive Care Unit**	2,286	158	2,444
**Other specialities**	57,512	3,466	60,978
**Total**	62,881	3,705	66,586

Classified as ECDC NORMAL

Supplementary Figure S2: Distribution of treatment indications among gentamicin treated patients, Norway, 2015-2313 (n =3,705 patients)

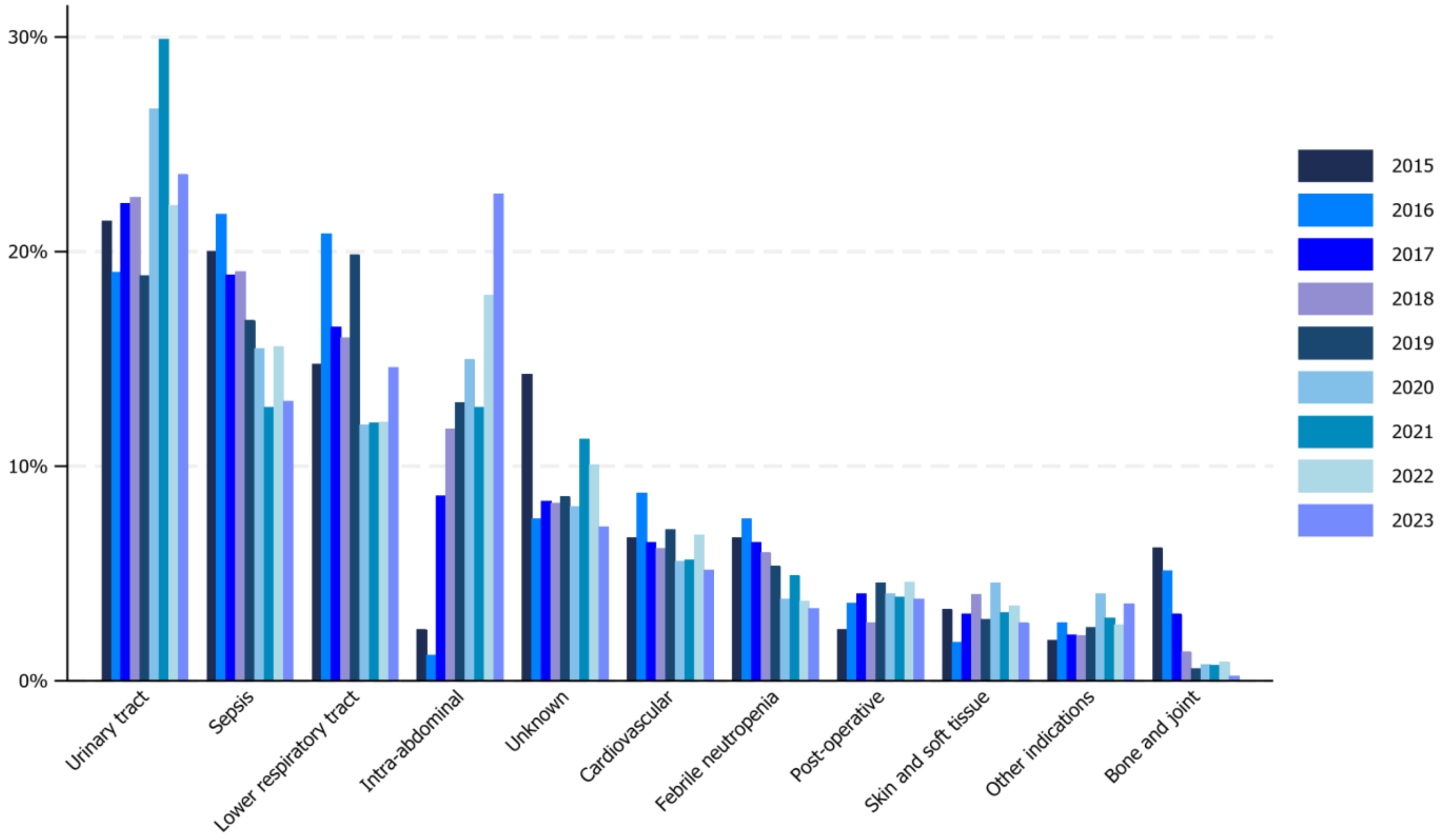

Urinary tract infections (lower and upper urinary tract infections).

Classified as ECDC NORMAL

Supplementary Table S8: Distribution of major antibiotic regimens per year, in urinary tract infections (n=9,083) (lower and upper UTI *)

	2015		2016		2017		2018		2019		2020		2021		2022		2023	
**Antibiotics**	**N**	**%**	**N**	**%**	**N**	**%**	**N**	**%**	**N**	**%**	**N**	**%**	**N**	**%**	**N**	**%**	**N**	**%**
**Pivmecillinam or Mecillinam**	212/766	27.7	258/1046	24.7	277/1092	25.4	262/1116	23.5	247/1097	22.5	201/916	21.9	198/964	20.5	197/1047	18.8	226/1039	21.8
**TMP/SMX or TMP**	115/766	15.0	178/1046	17.0	213/1092	19.5	230/1116	20.6	204/1097	18.6	182/916	19.9	174/964	18.0	195/1047	18.6	224/1039	21.6
**CTX, Cefuroxime or Ceftriaxone****	134/766	17.5	208/1046	19.9	187/1092	17.1	164/1116	14.7	204/1097	18.6	168/916	18.3	187/964	19.4	220/1047	21.0	182/1039	17.5
**Ampicillin or Amoxicillin**	86/766	11.2	111/1046	10.6	117/1092	10.7	117/1116	10.5	145/1097	13.2	97/916	10.6	114/964	11.8	142/1047	13.6	140/1039	13.5
**Ampicillin and Gentamicin**	27/766	3.5	38/1046	3.6	59/1092	5.4	85/1116	7.6	75/1097	6.8	75/916	8.2	102/964	10.6	87/1047	8.3	93/1039	9.0
**Other regimens and combinations*****	55/766	7.2	74/1046	7.1	71/1092	6.5	93/1116	8.3	70/1097	6.4	46/916	5.0	70/964	7.3	82/1047	7.8	64/1039	6.2
**Ciprofloxacin**	69/766	9.0	86/1046	8.2	90/1092	8.2	71/1116	6.4	80/1097	7.3	62/916	6.8	49/964	5.1	53/1047	5.1	43/1039	4.1
**Piperacillin-tazobactam**	35/766	4.6	48/1046	4.6	31/1092	2.8	38/1116	3.4	28/1097	2.6	32/916	3.5	33/964	3.4	37/1047	3.5	38/1039	3.7
**Other Gentamicin combinations******	18/766	2.3	25/1046	2.4	34/1092	3.1	32/1116	2.9	24/1097	2.2	30/916	3.3	20/964	2.1	14/1047	1.3	12/1039	1.2
**Meropenem or Ertapenem**	15/766	2.0	20/1046	1.9	13/1092	1.2	24/1116	2.2	20/1097	1.8	23/916	2.5	17/964	1.8	20/1047	1.9	17/1039	1.6

Abbreviations.: CTX: Cefotaxime; SMX: Sulfamethoxazole; TMP: Trimethoprim.
** Urinary tract infections (n=9,083): Upper (n=3,606) and lower (n=5,477) urinary tract infections.*
*** Detailed total distribution cephalosporins, CTX (n=1,352), Cefuroxime (n=179), Ceftriaxone (n=123)*
**** Other regimens and combinations: Benzylpenicillin, Phenoxymethylpenicillin, Vancomycin, Amoxicillin clavulanic acid, Cloxacillin, Ceftazidime, Dicloxacillin, Clindamycin, Cefotaxime and Ampicillin in combination, Cefazolin. These regimens may not be in accordance with national guidelines but reflect prescribing as recorded in the point prevalence survey and may represent targeted therapy.*
***** Other Gentamicin combinations: Gentamicin monotherapy, Gentamicin and Benzylpenicillin, Gentamicin and Cloxacillin, Gentamicin and Ampicillin and Metronidazole.*

Classified as ECDC NORMAL

Supplementary Table S9: Distribution of major antibiotic regimens per year, sepsis (n=3,987)

	2015		2016		2017		2018		2019		2020		2021		2022		2023	
**Antibiotics**	**N**	**%**	**N**	**%**	**N**	**%**	**N**	**%**	**N**	**%**	**N**	**%**	**N**	**%**	**N**	**%**	**N**	**%**
**CTX, Cefuroxime or Ceftriaxone** *	60/282	21.3	90/448	20.1	97/471	20.6	104/541	19.2	110/523	21.0	75/380	19.7	68/393	17.3	101/512	19.7	83/437	19.0
**Piperacillin-tazobactam**	44/282	15.6	69/448	15.4	56/471	11.9	87/541	16.1	67/523	12.8	58/380	15.3	73/393	18.6	88/512	17.2	73/437	16.7
**Other regimens and combinations* ***	54/282	19.1	70/448	15.6	71/471	15.1	84/541	15.5	67/523	12.8	53/380	13.9	51/393	13.0	60/512	11.7	55/437	12.6
**Major Gentamicin combinations*** **	31/282	11.0	50/448	11.2	67/471	14.2	79/541	14.6	68/523	13.0	43/380	11.3	43/393	10.9	56/512	10.9	55/437	12.6
**Cloxacillin or Dicloxacillin**	23/282	8.2	31/448	6.9	31/471	6.6	46/541	8.5	42/523	8.0	34/380	8.9	44/393	11.2	59/512	11.5	48/437	11.0
**Benzyl-/Phenoxymethylpenicillin**	14/282	5.0	41/448	9.2	36/471	7.6	26/541	4.8	37/523	7.1	23/380	6.1	31/393	7.9	34/512	6.6	40/437	9.2
**Amoxicillin or Ampicillin**	9/282	3.2	20/448	4.5	28/471	5.9	40/541	7.4	41/523	7.8	29/380	7.6	32/393	8.1	38/512	7.4	31/437	7.1
**Meropenem or Ertapenem**	16/282	5.7	28/448	6.3	32/471	6,8	21/541	3.9	25/523	4.8	28/380	7.4	19/393	4.8	23/512	4.5	21/437	4.8
**Other Gentamicin combinations********	11/282	3.9	22/448	4.9	12/471	2.5	20/541	3.7	20/523	3.8	18/380	4.7	9/393	2.3	15/512	2.9	3/437	0.7
**TMP/SMX or TMP**	7/282	2.5	5/448	1.1	17/471	3.6	11/541	2.0	24/523	4.6	15/380	3.9	16/393	4.1	17/512	3.3	14/437	3.2
**Ciprofloxacin**	9/282	3.2	18/448	4.0	11/471	2.3	15/541	2.8	18/523	3.4	2/380	0.5	5/393	1.3	14/512	2.7	10/437	2.3
**CTX and MDZ**	4/282	1.4	4/448	0.9	13/471	2.8	8/541	1.5	4/523	0.8	2/380	0.5	2/393	0.5	7/512	1.4	4/437	0.9

Abbreviations: CTX: Cefotaxime; MDZ: Metronidazole; SMX: Sulfamethoxazole; TMP: Trimethoprim.
** Detailed total distribution cephalosporins, CTX (n=670), Ceftriaxone (n=90), Cefuroxime (n=28)*
*** Other regimens and combinations: Dicloxacillin, Vancomycin, Amoxicillin, Clindamycin, Phenoxymethylpenicillin, Meropenem and Vancomycin, Ceftazidime, Benzylpenicillin and Clindamycin, Linezolid, Pivmecillinam. These regimens may not be in accordance with national guidelines but reflect prescribing as recorded in the point prevalence survey and may represent targeted therapy.*
**** Major gentamicin combinations: Benzylpenicillin and Gentamicin, Ampicillin and Gentamicin +/- Metronidazole.*
****Other Gentamicin combinations: Gentamicin monotherapy, Gentamicin and Cloxacillin.*

Classified as ECDC NORMAL

Supplementary Table S10: Distribution of major antibiotic regimens per year, lower respiratory tract infections (n=16,393)

	2015		2016		2017		2018		2019		2020		2021		2022		2023	
**Antibiotics**	**N**	**%**	**N**	**%**	**N**	**%**	**N**	**%**	**N**	**%**	**N**	**%**	**N**	**%**	**N**	**%**	**N**	**%**
**Benzyl-/Phenoxymethylpenicillin***	406/1265	32.1	601/1989	30.2	713/2162	33.0	791/2124	37.2	766/2162	35.4	506/1425	35.5	504/1639	30.8	553/1816	30.5	580/1811	32.0
**CTX, Cefuroxime or Ceftriaxone****	311/1265	24.6	486/1989	24.4	524/2162	24.2	432/2124	20.3	439/2162	20.3	322/1425	22.6	450/1639	27.5	473/1816	26.0	477/1811	26.3
**Amoxicillin or Ampicillin**	134/1265	10.6	221/1989	11.1	211/2162	9.8	220/2124	10.4	257/2162	11.9	135/1425	9.5	161/1639	9.8	166/1816	9.1	174/1811	9.6
**Other regimens and combinations*****	140/1265	11.1	182/1989	9.2	227/2162	10.5	193/2124	9.1	192/2162	8.9	124/1425	8.7	150/1639	9.2	190/1816	10.5	188/1811	10.4
**Piperacillin-tazobactam**	132/1265	10.4	238/1989	12.0	173/2162	8.0	164/2124	7.7	154/2162	7.1	124/1425	8.7	127/1639	7.7	159/1816	8.8	133/1811	7.3
**TMP/SMX or TMP**	30/1265	2.4	63/1989	3.2	100/2162	4.6	94/2124	4.4	97/2162	4.5	81/1425	5.7	89/1639	5.4	105/1816	5.8	101/1811	5.6
**Benzylpenicillin and Gentamicin**	23/1265	1.8	51/1989	2.6	51/2162	2.4	71/2124	3.3	84/2162	3.9	36/1425	2.5	42/1639	2.6	45/1816	2.5	55/1811	3.0
**Meropenem or Ertapenem**	29/1265	2.3	49/1989	2.5	38/2162	1.8	41/2124	1.9	38/2162	1.8	29/1425	2.0	52/1639	3.2	42/1816	2.3	30/1811	1.7
**Erythromycin, AZM or CAM**	30/1265	2.4	45/1989	2.3	58/2162	2.7	52/2124	2.4	54/2162	2.5	23/1425	1.6	25/1639	1.5	29/1816	1.6	31/1811	1.7
**Doxycycline**	10/1265	0.8	21/1989	1.1	34/2162	1.6	29/2124	1.4	31/2162	1.4	19/1425	1.3	16/1639	1.0	18/1816	1.0	18/1811	1.0
**Cefotaxime and MDZ**	12/1265	0.9	14/1989	0.7	15/2162	0.7	25/2124	1.2	30/2162	1.4	15/1425	1.1	16/1639	1.0	26/1816	1.4	14/1811	0.8
**Other Gentamicin combinations ********	8/1265	0.6	18/1989	0.9	18/2162	0.8	12/2124	0.6	20/2162	0.9	11/1425	0.8	7/1639	0.4	10/1816	0.6	10/1811	0.6

Abbreviations.: CTX: Cefotaxime; MDZ: Metronidazole; SMX: Sulfamethoxazole; TMP: Trimethoprim.
**Detailed total distribution Benzylpenicillin (n=4,663), Phenoxymethylpenicillin (n=757)*
*** Detailed total distribution CTX (n=3,423), Ceftriaxone (n=222), Cefuroxime (n=269)*
****Other regimens and combinations: Ciprofloxacin, Clindamycin, Amoxicillin clavulanic acid, Benzylpenicillin and Metronidazole, Cloxacillin, Ciprofloxacin and Clindamycin, Ceftazidime, Ciprofloxacin and Benzylpenicillin, Dicloxacillin. These regimens may not be in accordance with national guidelines but reflect prescribing as recorded in the point prevalence survey and may represent targeted therapy.*
*****Other Gentamicin combinations: Gentamicin and Ampicillin, Gentamicin monotherapy, Gentamicin and Cloxacillin, Gentamicin, Ampicillin and Metronidazole.*

Classified as ECDC NORMAL

Supplementary Table S11: Distribution of major antibiotic regimens per year, intra-abdominal infections (n=5,369)

	2015		2016		2017		2018		2019		2020		2021		2022		2023	
**Antibiotics**	**N**	**%**	**N**	**%**	**N**	**%**	**N**	**%**	**N**	**%**	**N**	**%**	**N**	**%**	**N**	**%**	**N**	**%**
**Piperacillin-tazobactam**	218/360	60.6	341/582	58.6	254/611	41.6	258/608	42.4	267/667	40.0	268/547	49.0	353/667	52.9	302/660	45.8	288/667	43.2
**Other regimens and combinations****	67/360	18.6	96/582	16.5	155/611	25.4	108/608	17.8	152/667	22.8	111/547	20.3	100/667	15.0	148/660	22.4	143/667	21.4
**TMP/SMX or TMP**	20/360	5.6	52/582	8.9	69/611	11.3	71/608	11.7	72/667	10.8	50/547	9.1	83/667	12.4	65/660	9,8	61/667	9.1
**Ampicillin, Gentamicin and MDZ**	0/360	0.0	2/582	0.3	18/611	2.9	44/608	7.2	56/667	8.4	41/547	7.5	40/667	6.0	66/660	10.0	89/667	13.3
**Ciprofloxacin**	23/360	6.4	34/582	5.8	30/611	4.9	31/608	5.1	23/667	3.4	19/547	3.5	25/667	3.7	13/660	2.0	14/667	2.1
**CTX, Cefuroxime or Ceftriaxone***	10/360	2.8	24/582	4.1	26/611	4.3	31/608	5.1	32/667	4.8	22/547	4.0	25/667	3.7	21/660	3.2	27/667	4.0
**Meropenem or Ertapenem**	10/360	2.8	25/582	4.3	18/611	2.9	23/608	3.8	22/667	3.3	10/547	1.8	19/667	2.8	22/660	3.3	22/667	3.3
**Cefotaxime and MDZ**	7/360	1.9	6/582	1.0	23/611	3.8	25/608	4.1	31/667	4.6	8/547	1.5	10/667	1.5	7/660	1.1	11/667	1.6
**Other Gentamicin combinations*****	5/360	1.4	2/582	0.3	18/611	2.9	17/608	2.8	12/667	1.8	18/547	3.3	12/667	1.8	16/660	2.4	12/667	1.8

Abbreviations.: CTX: Cefotaxime; MDZ: Metronidazole; SMX: Sulfamethoxazole; TMP: Trimethoprim.

**CTX (n=195), Ceftriaxone (n=16), Cefuroxime (n=7)*

***Other regimens and combinations: Ampicillin and Metronidazole, Amoxicillin, Ciprofloxacin and Metronidazole, Imipenem/cilastatin, Cefuroxime and Metronidazole, Metronidazole and Doxycycline, Ampicillin, Metronidazole and Trimethoprim-sulfametoxazole, Metronidazole, Piperacillin-tazobactam and Metronidazole.*

****Other Gentamicin combinations: Gentamicin and Ampicillin, Gentamicin monotherapy, Gentamicin and Benzylpenicillin, Gentamicin and Cloxacillin.*

Classified as ECDC NORMAL
